# Prevalence of skin manifestations in patients with COVID-19: a systematic review and meta-analysis

**DOI:** 10.3389/fmed.2024.1390775

**Published:** 2024-08-21

**Authors:** Beatriz Regina Lima de Aguiar, Elaine Barros Ferreira, Graziela De Luca Canto, Eliete Neves Silva Guerra, Paula Elaine Diniz dos Reis

**Affiliations:** ^1^Health Science Graduate Program, School of Health Sciences, University of Brasilia, Brasília, Brazil; ^2^Interdisciplinary Laboratory of Research Applied to Clinical Practice in Oncology, School of Health Sciences, University of Brasilia, Brasília, Brazil; ^3^Department of Dentistry, Brazilian Centre for Evidence-Based Research, Federal University of Santa Catarina, Florianopolis, Brazil; ^4^Laboratory of Oral Histopathology, School of Health Sciences, University of Brasilia, Brasília, Brazil

**Keywords:** systematic review, meta-analysis, COVID-19, skin manifestation, adult, aged

## Abstract

**Background:**

COVID-19 presents extrapulmonary manifestations that can aid in the diagnosis. Skin manifestations have been reported but their characteristics are not yet clear. Health professionals need information about its prevalence and main characteristics.

**Methods:**

This systematic review followed the PRISMA criteria. The protocol was registered in the PROSPERO (number CRD42020193173). Seven electronic databases and the gray literature were searched independently by two researchers. Observational analytical studies that presented data on the prevalence of skin manifestations in patients aged 19 or older with COVID-19 were included. Prevalence estimates were synthesized through a meta-analysis using random-effects models. Association meta-analysis and comparisons were performed for individual characteristics.

**Results:**

We included 31 studies with 10,934 patients, of which 10,121 tested positive for COVID-19. The general prevalence of skin manifestations was 29% (95% CI: 17.0–43.0; I^2^: 99%), the most in Africa, with a mean duration between 7 and 9 days and the most frequently affecting feet+hands (75%) and the trunk (71%). Patients with mild/moderate COVID-19 had more of chilblain-like+pernio-like lesions (97%) and inflammatory lesions (86%) than patients with severe or critical COVID-19. Manifestations of vascular origin were only in elderly patients and were significant with the severity of COVID-19 (*p* = 0).

**Conclusion:**

The global prevalence of skin manifestations is similar to other signs and symptoms of COVID-19. Skin assessment should be considered when investigating and diagnosing COVID-19 in adult and elderly patients.

**Systematic review registration**: PROSPERO, identifier CRD42020193173, https://www.crd.york.ac.uk/prospero/display_record.php?ID=CRD42020193173.

## 1 Introduction

In December 2019, a global pandemic emerged caused by the severe acute respiratory syndrome coronavirus-2 (SARS-CoV-2), called COVID-19 (1, 2). The most common symptoms are fever, dry cough, and, in some cases, shortness of breath (3, 4). These main symptoms are associated with SARS-CoV-2 virus affinity for cells in the respiratory tract (5). Age is a recognized risk factor for SARS-CoV-2 infection. It is estimated that patients over 50 years old are more susceptible to the virus and are more likely to develop severe manifestations of COVID-19 disease (5–7) As a result, early detection of signs and symptoms, along with laboratory diagnosis, is essential for disease management and controlling the spread of SARS-CoV-2 (8).

Various extrapulmonary symptoms have been documented in the literature, including anosmia, dysgeusia, headache, acute kidney injury, diarrhea, nausea, vomiting, cardiac injury, and skin manifestations (9–12). Skin manifestations were initially reported in both a Chinese and an Italian study, showing a prevalence of 1 and 20%, respectively (13, 14). These reported skin lesions ranged from erythematous rash to widespread urticarial, and chickenpox-like vesicles (14). Subsequent studies continue to report on these skin-related findings. Several studies describe possible mechanisms underlying the appearance of skin manifestations (15, 16). Even though the etiopathogenic mechanisms behind these skin symptoms remain speculative, deepening our understanding of them is essential (17). Health professionals need insights into the prevalence of skin manifestations, their main characteristics, onset timing, duration, and related factors to manage the co-manifestations related to COVID-19.

This systematic review aims to provide a thorough and up-to-date summary of skin manifestations in adult and elderly patients infected with SARS-CoV-2, reviewing all relevant observational studies to answer the following questions: What is the prevalence of skin manifestations in adult and elderly patients with confirmed COVID-19? What are the main characteristics of these skin manifestations? Are there demographic or disease-related factors that may be associated with developing skin manifestations of COVID-19?

## 2 Methods

This systematic review was conducted following the Preferred Reporting Items for Systematic Reviews and Meta-Analyses (PRISMA) checklist (18). The protocol was registered in the International Prospective Register of Systematic Reviews (PROSPERO) (number CRD42020193173) (19). The research question as well as the eligibility criteria were defined following the acronym PEOS (Population, Exposition, Outcomes, and Study design), being: (P) adult or elderly patients; (E) SARS-CoV-2 infection with a positive laboratory test; (O) frequency data of skin manifestations; and (S) observational analytical studies. We included only observational studies because they had greater evidence.

### 2.1 Eligibility criteria

We considered those observational analytical studies eligible for this systematic review that presented frequency data of skin manifestations in adult or elderly patients exposed to SARS-CoV-2 infection with a positive laboratory test. We excluded studies for the following reasons: (1) studies evaluating skin manifestations of COVID-19 in individuals under 19 years of age (children and adolescents), (2) studies that did not report whether adult patients had a positive diagnosis, confirmed by polymerase chain reaction (PCR) test, serology test, or antigen test, for COVID-19, (3) studies in which adult patients had a negative PCR, serology, or antigen test for COVID-19; (4) studies that did not individualize data for adult patients with a confirmed diagnosis of COVID-19 by laboratory test; (5) patients with skin manifestations related to diseases other than COVID-19; (6) patients with only skin manifestations of severe vasculopathies (vaso occlusive); (7) studies that did not individualize results of skin manifestations of COVID-19 for adult or elderly patients (mixed samples); (8) studies that reported skin manifestations associated with adverse vaccine reactions; (9) studies that reported skin manifestations associated with other infections than SARS-CoV-2 (10) studies that reported skin manifestations associated with adverse drug reactions; (11) clinical trials, reviews, book chapters, letters, personal opinions, conference abstracts, case reports, and case series; (12) studies that did not report sufficient information; (13) studies in languages that do not use the Latin-Roman alphabet, and (14) skin manifestations of COVID-19 after 3 months of diagnosis.

### 2.2 Information sources and search strategy

The search strategy was elaborated and adapted for each electronic database: CINAHL, EMBASE, LILACS, LIVIVO, PubMed, Scopus, and Web of Science Core Collection. In addition, a gray literature search was conducted on Google Scholar and ProQuest Dissertations & Thesis Global. The search strategy is shown in Supplementary Table S1.

The search was performed on June 6, 2023, in all databases and gray literature. The software reference manager (EndNote X7, Thomson Reuters, Philadelphia, PA) was used to collect references and remove duplicate articles. Hand searches of reference lists from the included studies were also carried out. No time or language restrictions were applied.

### 2.3 Study selection

The selection was completed in two phases. In Phase 1, two reviewers (B.R.L.A. and E.B.F.) independently reviewed the titles and abstracts of all identified electronic database citations using the *Rayyan^®^* software (20). A third author (P.E.D.R.) was involved when required to make a final decision. Any studies that appeared to not fulfill the inclusion criteria were discarded. In Phase 2, the same selection criteria were applied to the full articles to confirm their eligibility. The same two reviewers (B.R.L.A. and E.B.F.) independently participated in Phase 2. The reference lists of all included articles were also reviewed. Both examiners read the selected articles. Any disagreement in either phase was resolved by discussion and mutual agreement between the three reviewers. The final selection was always based on the full text of the publication.

### 2.4 Data collection process and items

Data were extracted from study documents, including information about study characteristics (author(s), country, year of publication, design, and data collection period), population characteristics (sample size, sex, age, proportion of positive and negative patients for COVID-19, laboratory test performed, signs and symptoms of COVID-19, and severity of COVID-19), and outcome characteristics (number of patients with skin manifestations, type of skin manifestations, morphological characteristics, location, duration, period of onset, other associated skin symptoms, skin biopsy, and previous skin injury). Two reviewers (B.R.L.A. and E.B.F.) independently extracted data, and a third reviewer (P.E.D.R.) resolved disagreements, if any. The study authors were contacted for unreported data or additional details.

### 2.5 Risk of bias assessment

The risk of bias in the included studies was assessed using the Critical Appraisal Checklist for Prevalence Studies tool from the Joanna Briggs Institute (JBI) (21). The first and second reviewers (B.R.L.A. and E.B.F.) evaluated the risk of bias independently, and any disagreement was resolved by consensus with the third reviewer (P.E.D.R.) for the final decision. The risk of bias assessment tool questions were answered with “yes,” “no,” “unclear,” or “not applicable.” We calculated the proportion of “yes” answers for each question in the instrument to assess the most prevalent biases concerning the studies’ reporting.

### 2.6 Effect measures

The primary outcome was the proportion of skin manifestations in adults and elderly patients with any laboratory test positive for COVID-19 and the estimation of 95% Confidence Intervals (95% CI). The following subgroup analyses were performed: (1) Prevalence of skin manifestations by continent in the world, (2) Prevalence by type of skin manifestation, (3) Prevalence of skin manifestations by morphological characteristics, (4) Prevalence by location of skin manifestation (part of the body), and (5) Individual and clinical characteristics of patients presenting with skin manifestations of COVID-19. The secondary outcome was the association of skin manifestations with (1) female and male patients and (2) the severity of COVID-19.

### 2.7 Synthesis methods

We performed a qualitative synthesis of the main characteristics of the included studies. Individual and clinical characteristics of the patients were summarized based on the assessment of the proportion of cases and estimation of 95% confidence intervals (95% CI) using the OpenEpi online software (22). Prevalence meta-analysis was performed using Meta-XL^®^ 5.3 add-in Microsoft Excel software with random effect. Forest plots of the prevalence analysis will be expressed by relative or absolute frequencies and their 95% CI. Association analysis was performed using the Cochrane Collaboration Review Manager^®^ 5.4 software with odds ratio (OR) and 95% CI on dichotomous variables. Heterogeneity will be evaluated by the inconsistency index (I^2^), estimation of the variance of real effects (Tau^2^), and Cochran’s Q significance level of 5% (Chi^2^). Chi^2^ test evaluated the association between the severity of COVID-19 and the type of skin manifestations. To be faithful to the results presented in the primary studies, we did not use data transformation tools.

### 2.8 Assessment of the certainty of the evidence

For the association outcomes, we used the Grading of Recommendation, Rating, Development, and Evaluation (GRADE) criteria (23) to assess the certainty of the evidence. GRADEpro was used to build the summary of the findings table. All evaluations were conducted by the first and second reviewers (B.R.L.A. and E.B.F.) independently and a third reviewer resolved any disagreements. The certainty of the evidence was expressed as high, moderate, low, or very low.

## 3 Results

### 3.1 Study selection

**Figure 1:**
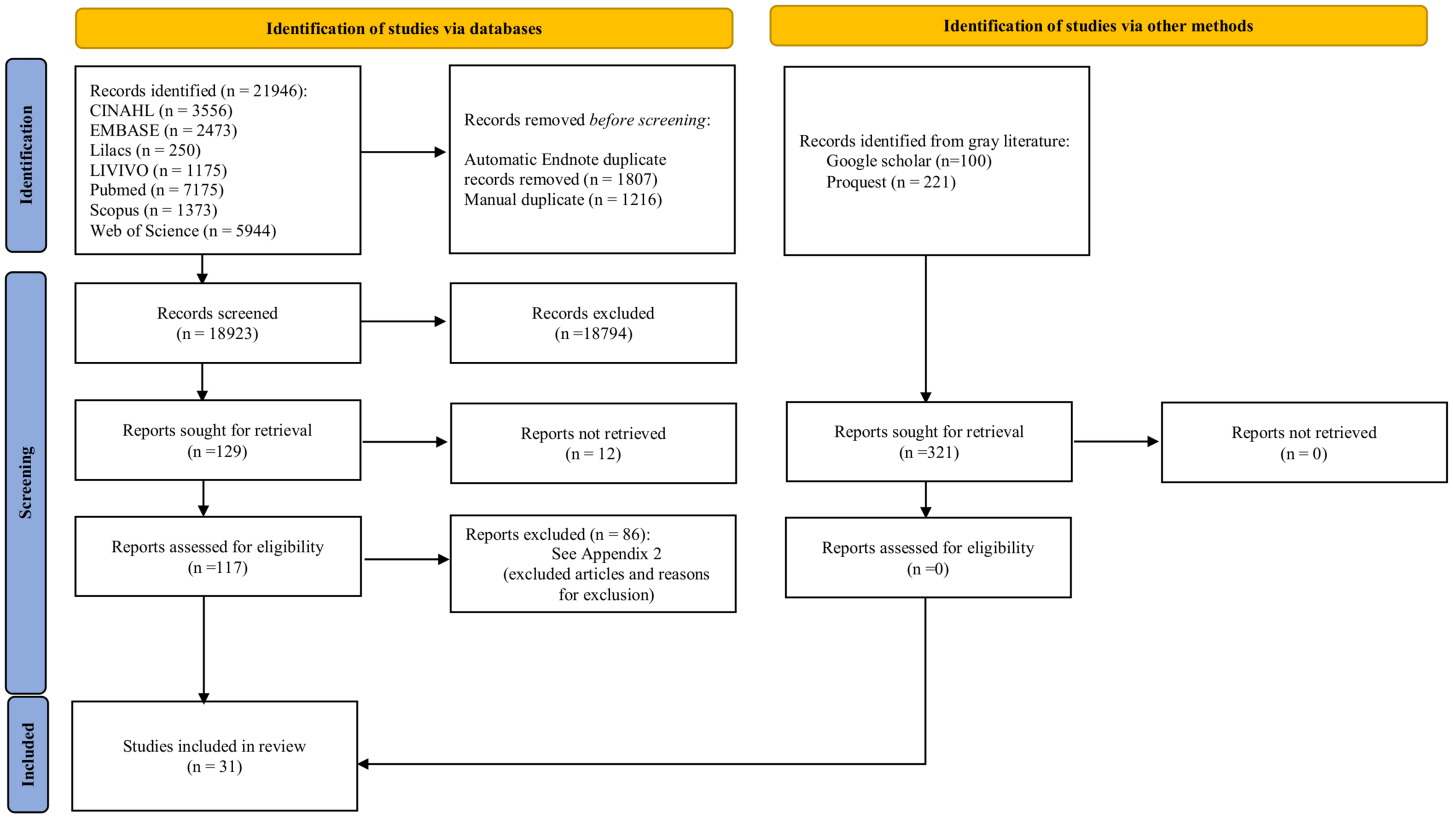
Flow diagram of search and selection process. Adapted from Page et al. (18).

Searching the databases resulted in 18,923 references after removing duplicates. Among these studies, 18,794 were excluded after reading the titles and abstracts. A 129 studies were selected to be read in full text, and 12 studies could not be retrieved. We contacted authors via email and through the ResearchGate platform to retrieve the missing article, but to this date, we have not received a response. Thus, 117 studies were read in full text. Eighty-six studies were excluded following the eligibility criteria. The reasons for exclusion can be found in Supplementary Table S2. Thus, 31 observational studies were included in this systematic review (Figure 1) (24–54).

### 3.2 Study characteristics

Among the 31 studies included in this review, two were retrospective cohorts (31, 48), 11 were prospective cohorts (24, 26, 30, 33, 34, 36–38, 43, 51, 54), and 18 were cross-sectional (25, 27–29, 32, 35, 39–42, 44–47, 49, 50, 53, 54). About the geographic location of the studies, 42% were performed in Asia, 29% in Europe, 13% in Latin America, 3% in North America, 3% in Africa, 6% were performed in two continents [Africa and Asia (47), and Asia and Europe (27)], and 3% (one study) was performed simultaneously in North America, Europe, Asia, Latin and the Caribbean, Africa, and Oceania (29). In all studies included in this review, patients were selected and evaluated between January 2020 and August 2021. The total sample of this review was 10,934 patients, of which 10,121 were positive for COVID-19 in some laboratory tests. Table 1 presents a summary of the main characteristics of the patients included in this review. Details about the individual characteristics of each study included in this review can be accessed in Supplementary Table S3.

**Table 1:** Summary of characteristics of patients (*n* = 10,934).

Characterization of COVID-19 patients (*n* = 10,934)				
AGE (years)				
Mean ± SD^a^	46.7 ± 9.4	Min–Max		20–96
		n	%	CI 95%
Sex				
Male		2,942	27	26.1–27.8
Female		2,744	25	24.3–25.9
N/A		5,248	48	47.1–48.9
Laboratory test COVID-19^b^				
PCR		10,466	96	95.3–96.1
Serology (IgG)		1,066	10	9.2–10.3
Serology (IgM)		1,036	10	8.9–10.0
Serology (NI)		75	1	0.5–0.9
Serology (IgA)		58	1	0.4–0.7
N/A		468	4	3.9–4.7
Result of COVID-19 test				
Positive^c^		10,121	93	92.1–93.0
Negative		813	7	7.0–7.9
Symptoms of COVID-19				
Not informed		8,178	75	74.0–75.6
Symptomatic^d^		2,476	23	21.9–23.4
	Flulike symptoms (myalgia, headache and/or asthenia)	1,015	41	39.1–42.9
	Fever	912	37	35.0–38.8
	Cough	679	27	25.7–29.2
	Anosmia and/or Ageusia	527	21	19.7–22.9
	Dyspnea	364	15	13.4–16.2
	Gastrointestinal symptoms (nausea, vomiting, diarrhea and/or abdominal pain)	344	14	12.6–15.3
	Lower respiratory tract symptoms (pneumonia, chest pain or other)	278	11	10.0–12.5
	Sore throat	133	5	4.6–6.3
	Upper respiratory tract symptoms (nasal itching, rhinorrhea or other)	123	5	4.2–5.9
	Not specified	246	10	8.8–11.2
Asymptomatic		280	3	2.3–2.9
Severity of COVID-19				
Mild/Moderate		3,295	30	29.3–31.0
Severe/Critical		718	7	6.1–7.0
Asymptomatic		280	3	2.3–2.9
Not Specified		6,641	61	59.8–61.7

95% CI, 95% confidence interval; PCR, polymerase chain reaction; IgG, immunoglobulins G; IgM, immunoglobulin M; IgA, immunoglobulin A; N/A, not addressed.

a Nineteen studies report the mean age (*n* = 4,946).

b Patients could undergo more than one test for COVID-19.

c Patients with a positive result in any laboratory test for COVID-19.

d The percentage of each individual symptom was calculated based on the number of symptomatic patients (*n* = 2,476).

Nineteen studies reported the mean age of the patients (24, 26, 31–35, 39, 41–43, 45–50, 53, 54). The overall mean age was 46.7 ± 9.4 years, ranging from 20 to 96 years. Most studies did not report the number of patients by female/male sex. Among those who reported, the proportion of female and male patients was similar.

All the included studies reported patients with confirmed COVID-19 infection, most of them used PCR, except for 2 studies (25, 51) which accounts for 468 patients out of the total patients that did not report the method of diagnosis. Additionally, five studies also used serological tests for diagnostic testing (29, 30, 32, 36, 37).

We grouped the severity of COVID-19 according to the World Health Organization (WHO) criteria (55). Patients with mild and moderate COVID-19 may develop pneumonia and require hospitalization but do not require oxygen support. Patients with severe and critical COVID-19 require non-invasive or invasive oxygen support and may require intensive care. Most studies did not report the signs and symptoms of COVID-19 (75%) or the severity of the disease (61%). Among those who reported, 3% were asymptomatic, the majority developed flulike symptoms, fever, and cough, and were diagnosed with mild/moderate COVID-19.

### 3.3 Risk of bias in studies

Articles meeting the inclusion criteria were critically appraised for prevalence studies (Supplementary Table S4). The most significant concerns for these studies were whether the study participants were sampled appropriately (Q2) and whether the sample size was adequate (Q3). Most studies did not report aspects of the sample regarding the inclusion process or sample calculation. In these cases, they were evaluated as unclear. Additionally, some studies did not apply similar methods for evaluating skin reactions because they involved different evaluators or even considered the patient’s own evaluation (Q6 and Q7).

### 3.4 Synthesis of results

Six studies included patients with skin manifestations suspected of COVID-19 and carried out testing (24, 25, 29, 30, 36, 39). Although some patients were negative in laboratory testing (*n* = 813), they were strongly suspected of COVID-19 by computed tomography and suggestive signs and symptoms. Therefore, in this review, we describe the skin manifestations presented by these patients in a group of patients negative for COVID-19. Supplementary Table S5 shows the absolute frequency of the patients who were positive and negative for COVID-19 in some laboratory tests; the main characteristics of the skin manifestations are presented. The skin manifestation analysis in this review considers only those found in patients with a positive laboratory test for COVID-19.

#### 3.4.1 Prevalence of skin manifestations in COVID-19 patients

**Figure 2:**
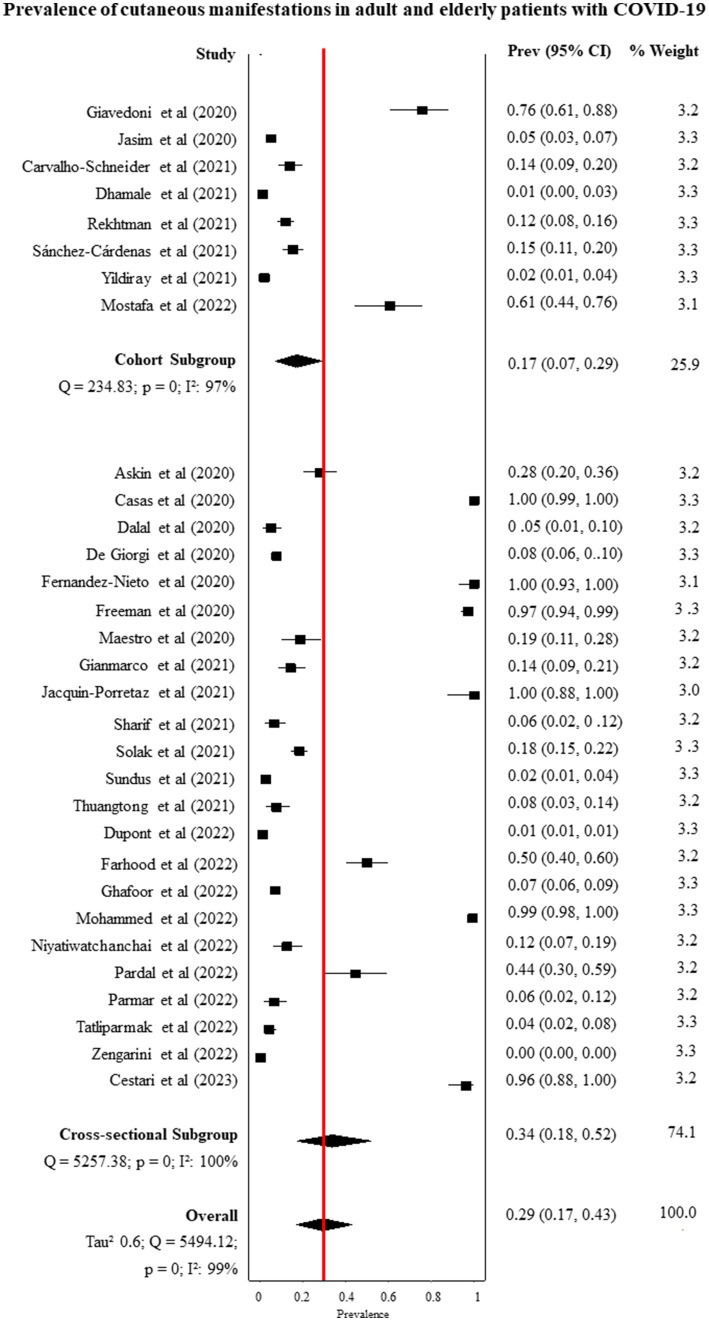
General prevalence of skin manifestations in adults and elderly patients with a positive laboratory test for COVID-19 and by type of study (*p* < 0.05 for all prevalence data). 95% CI, 95% confidence interval; I^2^, inconsistency index.

**Figure 3:**
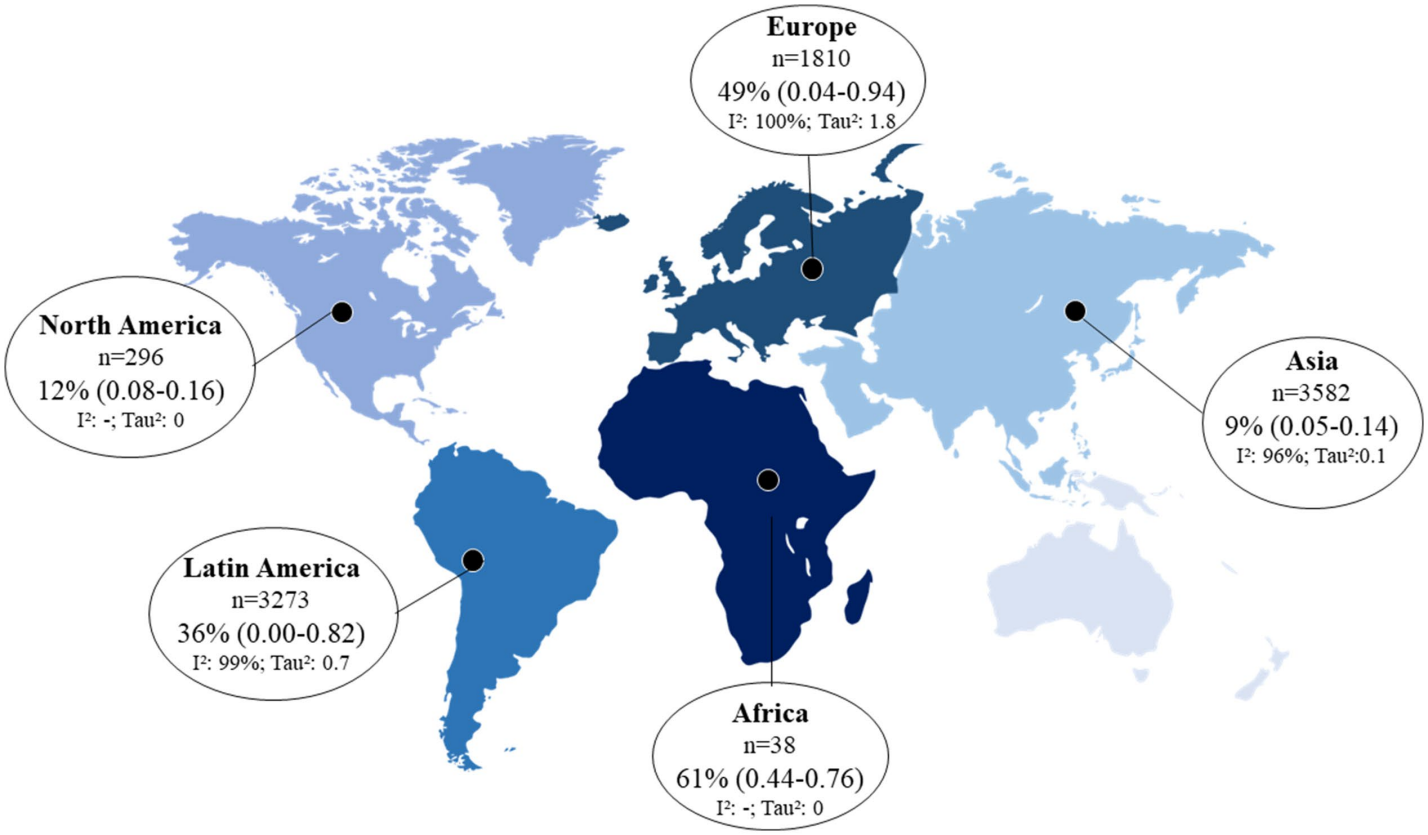
Prevalence of skin manifestations in adults and elderly patients with a positive laboratory test for COVID-19 by geographic region (*p* < 0.05 for all prevalence data). 95% CI, 95% confidence interval; I^2^, inconsistency index. *The prevalence of skin manifestations for each geographic region was calculated considering the total number of patients positive for COVID-19 per continent (n value shown in the figure); prevalences that did not present an I^2^-value were calculated from only one study.

All studies presented sufficient data for proportion meta-analysis. The prevalence of skin manifestations in the cohort and cross-sectional studies was 17% (95% CI: 7.0–29.0) and 34 (95% CI: 18.0–52.0), respectively. The overall prevalence was 29% (95% CI: 17.0–43.0; I^2^: 99%, *p* = 0), and the funnel plot showed that there is publication bias due to a large dispersion of data from the studies included in the meta-analysis (Figure 2; Supplementary Figure S1). In an assessment of the prevalence of skin manifestations by continent in the world, three studies were excluded from the analysis because they were carried out on more than one continent (27, 29, 47). Africa had the highest prevalence (61%), followed by Europe (49%), Latin America (36%), North America (12%), and Asia (9%). Figure 3 presents the prevalence of skin manifestations in patients with a positive laboratory test for COVID-19 by continent. All proportions showed high heterogeneity.

#### 3.4.2 Summary of characteristics of skin manifestations in COVID-19 patients

**Figure 4:**
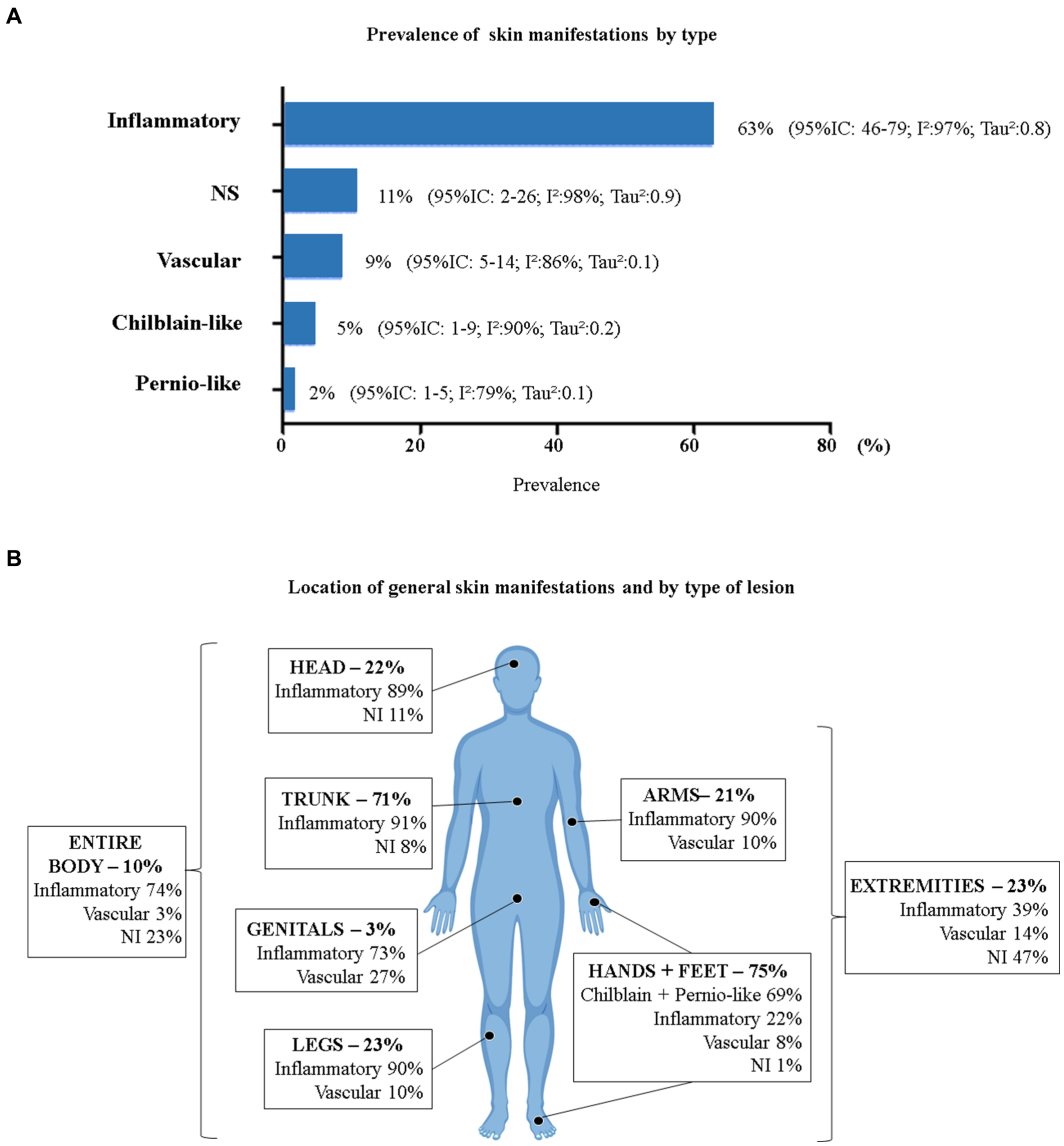
Prevalence of COVID-19 skin manifestations by type. **(A)** General prevalence of skin manifestations (*p* < 0.05 for all prevalence data). **(B)** General prevalence of skin manifestations by body area and type of injury (*n* = 383). 95% CI, 95% confidence interval; I^2^, inconsistency index; NS, Not Specified. *Head = head and face; Trunk = neck, chest, back, abdomen, and/or hips; Hands + Feet = palms, soles, hands, feet, and/or fingers; Entire Body = disseminated lesions (more than 2 body segments); Extremities = arms, legs, hands, and feet. (Body image taken from free-access Vecteezy resources).

Only two studies did not report the characteristics of the skin manifestations (24, 33). Considering the total number of skin manifestations developed by patients who tested positive for COVID-19 (*n* = 1,343), we evaluated the relative frequency by lesion characteristics using a proportion meta-analysis (Figure 4A). Inflammatory manifestations were the most prevalent (63%) and were all classified as rashes. Lesions of vascular origin occurred in 9% of cases, followed by chilblain-like lesions (5%) and pernio-like lesions (2%). All proportions showed high heterogeneity (I^2^ > 78% and *p* = 0).

Ten studies presented data on the location of cutaneous manifestations (*n* = 383) (28, 29, 31, 34, 37, 39, 40, 43, 51, 54). Figure 4B shows the location of skin manifestations by body area and type of injury. Patients could present with more than one type of injury in more than one area of the body. The most affected areas were the feet and hands (75%) and the trunk (71%).

The type of skin manifestation varied in frequency for each location on the body. Inflammatory lesions were the most prevalent in all areas of the body, except for the hands and feet, where the most prevalent were chilblain-like and pernio-like lesions. All chilblain-like and pernio-like lesions were found in the hands and feet. Inflammatory lesions were most common on the trunk (91%), followed by the arms and legs (90% each) and head (89%). Vascular manifestations on the skin were more frequent in the genital areas (27%) and in the extremities of the body (14%).

**Figure 5:**
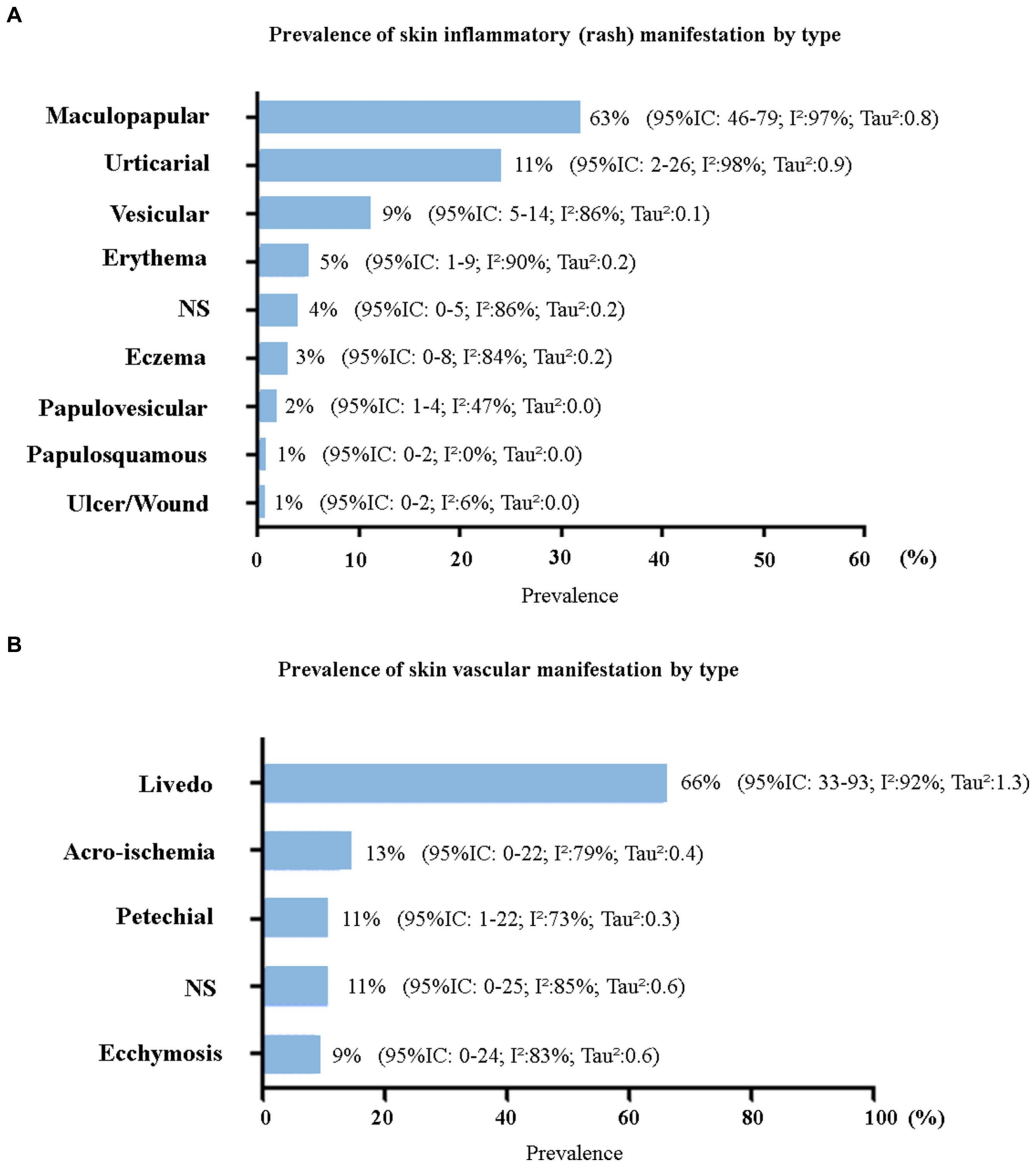
Prevalence of inflammatory and vascular skin manifestations by morphological type. **(A)** Inflammatory manifestations (rash) (*n* = 758). **(B)** Vascular manifestations (*n* = 131). 95% CI, 95% confidence interval; I^2^, inconsistency index; NS, not specified.

The total sample of inflammatory skin manifestations (rash) was 758 patients, and the sample of vascular skin manifestations was 131 patients. Figure 5 shows the prevalence by morphological type of lesion. Among the patients who presented with a rash, the majority (63%; 95% CI: 46.0–79.0, I^2^: 97% *p* = 0, Tau^2^: 0.8) presented with a maculopapular morphology, also called morbilliform. Urticaria was present in 11% of the sample. Vesicular lesions also included pustules and other bullous lesions, accounting for 9%. Erythematous lesions corresponded to 5% and varied from erythema-type macular, nodosum, elevatum, targetoid, multiform, and other types.

Among patients who presented with cutaneous manifestations of vascular origin, the majority presented with a livedoid lesion (66%; 95% CI: 33.0–93.0, I^2^: 92% *p* = 0, Tau^2^: 1.2), which also included purpura and/or necrosis. Acro-ischemic injuries were the second most prevalent (13%). More details about the type of skin manifestation can be accessed in Supplementary Table S5.

Regarding the characteristics of the reported skin manifestations, Table 2 presents a summary of the type of lesion and the overall total. The sample evaluating the general average age and duration of skin manifestations varied concerning to the total because only eight studies (25, 31, 38, 39, 46–48, 50) and five studies (25, 27, 31, 45, 46), respectively, presented these individualized data. Both variables were presented as the mean and standard deviation of the means reported in each study. Studies that presented only a median age and the median duration of the lesions were excluded from this analysis.

**Table 2:** Summary of characteristics of skin manifestation in patients with COVID-19 confirmed (*n* = 1,343).

	Chilblain-like (*n* = 159)		Pernio-like (*n* = 50)		Inflammatory (rash) (*n* = 758)		Vascular (*n* = 131)		Total (*n* = 1,343)	
Age (years)^a^										
Mean ± SD	30.4 ± 10.2		N/A		40.9 ± 12.2		67.2 ± 6. 0.3		44.3 ± 9.3	
Min–Max	21–45				20–61		60–76		20–76	
Duration (days)^b^										
Mean ± SD	8.5 ± 4.2		N/A		8.3 ± 4.0		8.9 ± 5.0		7.5 ± 1.0	
Min–Max	5–12				3–16		4–14		3–16	
	%	95% CI	%	95% CI	%	95% CI	%	95% CI	%	95% CI
Onset^c^										
Skin previous	10	6.3–15.7	10	4.3–21.4	7	5.5–9.2	3	1.2–7.6	9	7.9–11.0
Same time	31	24.2–38.4	6	2.1–16.2	44	40.3–47.4	39	31.0–47.5	43	40.1–45.3
Skin after	52	43.9–59.2	30	19.1–43.8	35	31.9–38.7	16	10.7–23.3	34	31.5–36.5
Not reported	8	4.4–12.7	54	40.4–67.0	14	11.6–16.5	42	33.9–50.6	14	12.3–16.0
Skin symptoms										
Pain and/or burning	8	4.4–12.7	44	31.2–57.7	8	6.0–9.8	2	0.8–6.5	7	6.2–9.0
Itching	6	3.0–10.4	22	12.8–35.2	38	34.4–41.2	3	1.2–7.6	30	27.1–32.0
Asymptomatic	-		6	2.1–16.2	3	2.4–5.0	8	4.8–14.4	5	3.9–6.3
Not reported	87	80.7–91.2	28	17.5–41.7	51	47.6–54.7	86	79.3–91.1	58	55.4–60.7
Previous skin injury										
Reported	.	..	..	..	..	..	..	..	4	2.7–4.7
Skin biopsy										
Sample collected for biopsy	..	..	..	..	..	..	..	..	7	5.6–8.2
COVID-19 tested^d^	..	..	..	..	..	..	..	..	4	1.7–10.8
Positive	..	..	..	..	..	..	..	..	0	–

95% CI, 95% confidence interval; N/A, not addressed.

a Eight studies report the mean age (*n* = 677).

b Five studies report the mean age (*n* = 427).

c Timing to appearance of the skin manifestations with respect to other symptoms.

d Relative frequency calculated based on the total number of samples collected for biopsy (4/91).

The general mean age of patients with a positive laboratory test for COVID-19 and who presented with skin manifestations was 44.3 ± 9.3 years, ranging from 20 to 76 years. Chilblain-like lesions were present in young adult patients (mean age 30.4 SD ± 10.2 years old), and inflammatory lesions (rash) were present in patients around 40.9 ± 12.2 (ranging from 20 to 61 years old). Lesions of vascular origin were present in patients over 60 years of age (mean: 67.2 ± 6.3, 6–76). The mean duration of skin manifestations was similar between the groups.

Regarding the onset of the skin manifestations, chilblain-like and pernio-like lesions appeared more frequently after resolution of other COVID-19 symptoms or 10 days after diagnosis (52 and 30%, respectively). Inflammatory and vascular lesions appeared most frequently alongside other COVID-19 symptoms or along with the diagnosis (44 and 39%, respectively). Considering the total number of skin manifestations, approximately 9% appeared before the onset of other COVID-19 symptoms.

Eight articles presented the frequency of symptoms associated with skin manifestations (25, 28, 34, 40, 43, 46, 50, 54). Itching was the most common symptom (30%) and was more common in inflammatory and pernio-like lesions. Pain and/or burning were more common in pernio-like lesions (44%). No other symptoms were reported.

Previous skin injuries were reported by 12 studies: six studies reported that no patient in the sample had previous skin injury (26, 35, 39, 41, 45, 52), and other six studies reported previous skin injury (28, 29, 40, 42, 48, 51). The types of injury reported were atopic dermatitis, psoriasis, chronic urticaria, alopecia areata, melanoma, and hidradenitis suppurativa, with a frequency of 4%.

Regarding skin sample collection for biopsy, three studies reported that they did not perform a biopsy (31, 34, 54). Five studies performed skin biopsy, totaling 91 patients (28–30, 36, 38). Only one study tested COVID-19 on skin samples from 4 patients, and all were negative (28).

#### 3.4.3 Association between skin manifestations and the characteristics of COVID-19 patients

**Figure 6:**
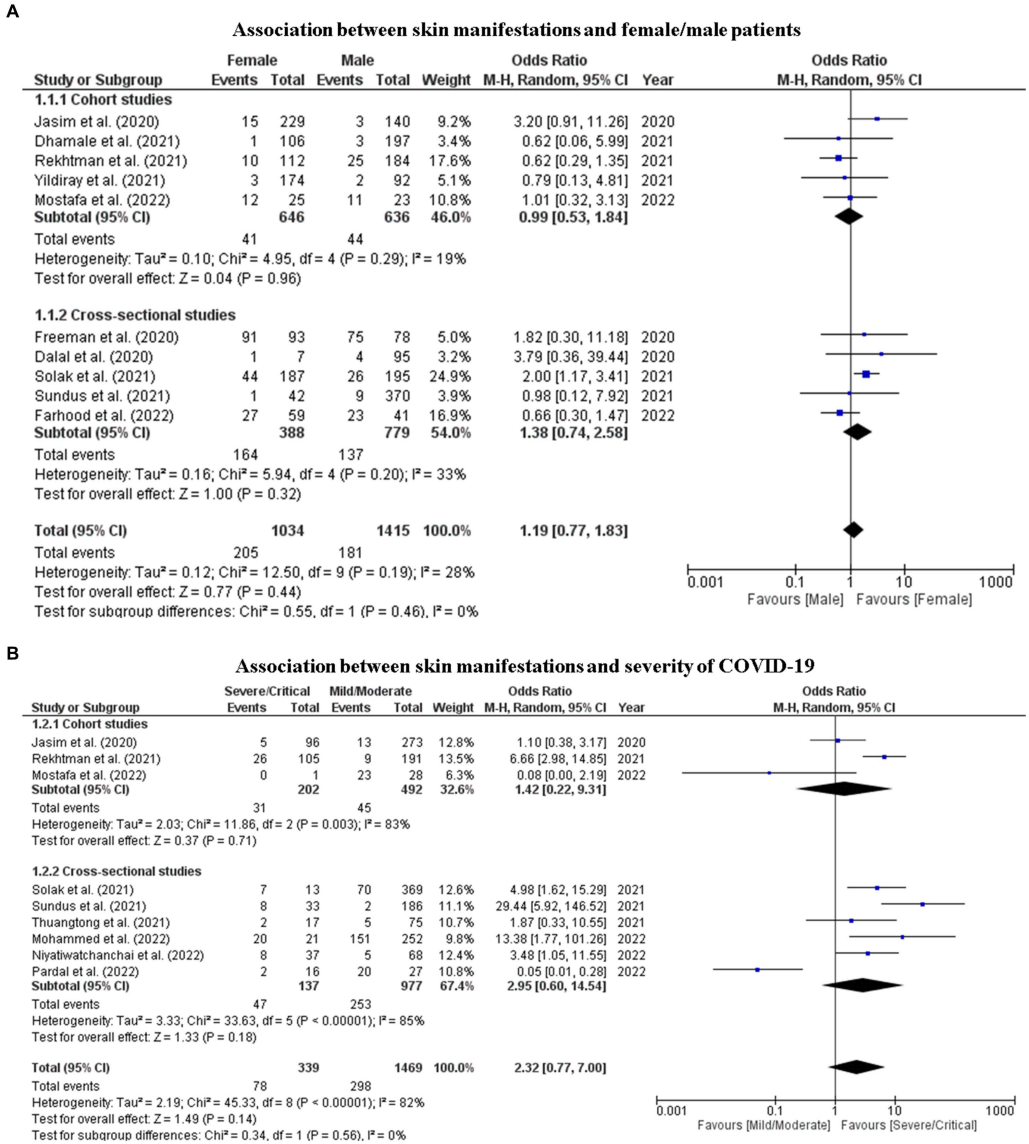
Forest plot of the association analysis of the occurrence of skin manifestations among patients with a positive diagnostic test about **(A)** sex (female or male) and **(B)** severity of COVID-19 (mild/moderate or severe/critical). Chi^2^, chi-square test; I^2^, inconsistency index; M-H, Mantel–Haenszel Test; OR, odds ratio; Q, Cochran’s Q test; Z, *Z*-test.

Random-effects meta-analysis showed that there was no statistically significant association between female and male and the occurrence of skin manifestations of COVID-19 (OR = 1.19; 95% CI: 0.8–1.8, I^2^: 28%, *p* = 0.44) (Figure 6A), nor between the severity of COVID-19 and the occurrence of skin manifestations (OR = 2.32; 95% CI: 0.8–7.0, I^2^: 82%, *p* = 0.14) (Figure 6B). The funnel plot showed homogeneity between data on skin manifestations by sex, demonstrating a low probability of publication bias (Supplementary Figure S2).

**Figure 7:**
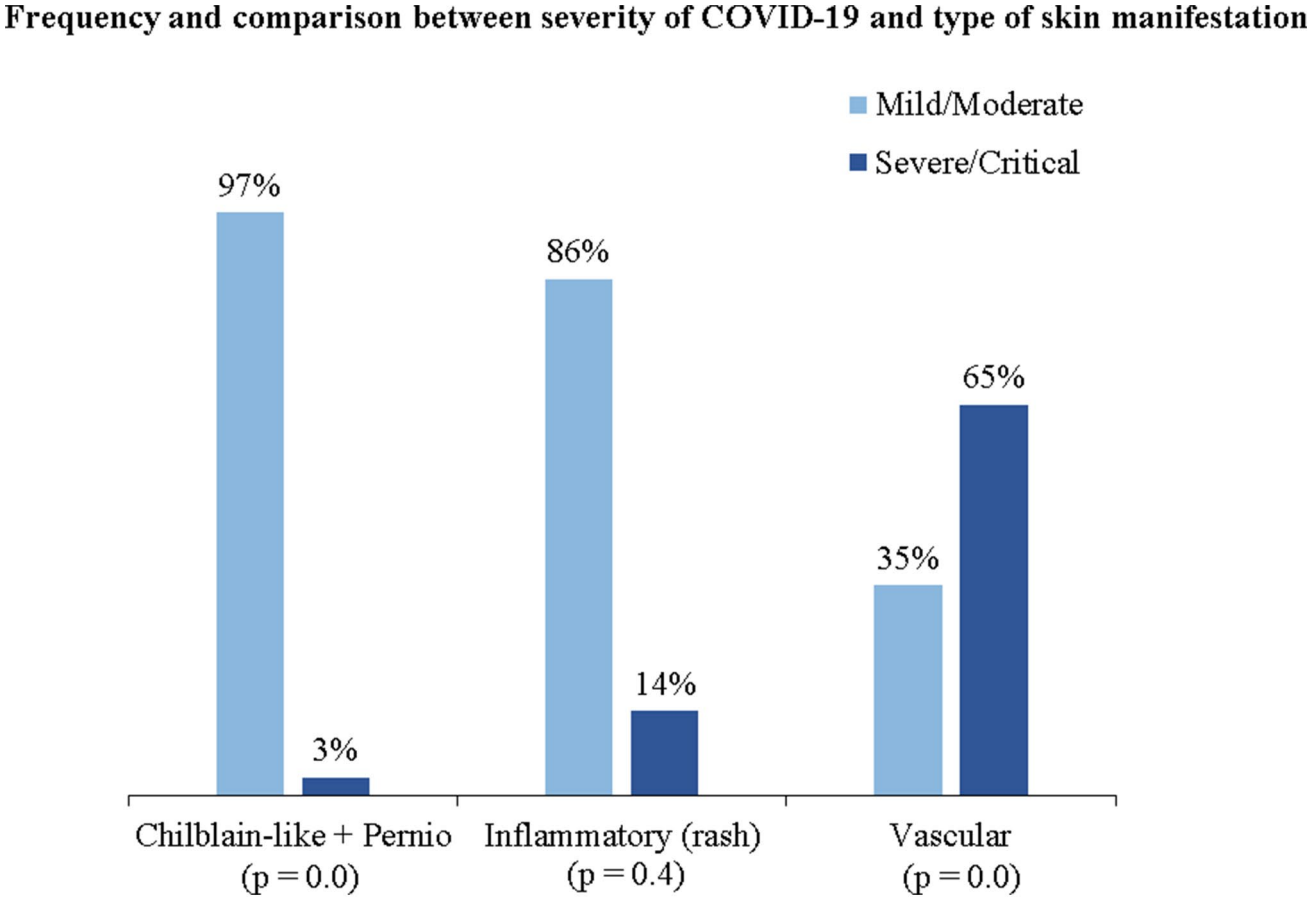
Proportion and comparison between the severity of COVID-19 and the type of skin manifestation presented. *The *p*-value was based on the chi-square test (Chi^2^).

In the complementary analysis, we found an association between the severity of COVID-19 and the type of skin manifestation. We assessed the proportion of patients with moderate/mild and severe/critical COVID-19 who developed each type of skin manifestation (Figure 7).

Patients with mild/moderate COVID-19 had a higher proportion of chilblain-like + pernio-like lesions (97%; *p* = 0) and inflammatory lesions (86%; *p* > 0.05) than patients with severe/critical COVID-19. Skin manifestations of vascular origin were more frequent in patients with severe/critical COVID-19 (65%) than in patients with mild/moderate COVID-19 (35%), with a statistically significant value (*p* = 0).

### 3.5 Certainty of evidence

When assessing the risk of bias, several studies did not make it clear whether participants were adequately sampled, which may have impacted the selection of patients of different severity. Regarding females and males, there was a similar proportion between female and male participants. Therefore, we assume that sampling may not have impacted this variable. Additionally, there were serious problems due to inconsistency and imprecision in the assessment by female(s)/male(s) and very serious problems due to inconsistency in the assessment by the severity of COVID-19.

Therefore, there was very low certainty of evidence that there was no significant difference between the occurrence of skin manifestations of COVID-19 among females and males with the severity of COVID-19 (mild/moderate or severe/critical) (Supplementary Table S6). This suggests that our confidence in the estimated effect is limited.

## 4 Discussion

This systematic review included 10,121 patients aged 20 years or older who tested positive for COVID-19 in any laboratory test. Although we only included observational studies in this review, several case studies and case series have been published to date. These studies reported cutaneous manifestations of COVID-19 in adult and elderly patients, and most included patients without a confirmed diagnosis (56–59).

In the risk-of-bias analysis of the studies included in this systematic review, the main concerns were that the majority did not report how participants were selected and that some studies did not report how patients were evaluated. However, considering that the methodology of observational studies is better designed than that of descriptive studies, we believe that this review has greater evidence than reviews already carried out that have included descriptive studies and studies of various methodologies.

The general prevalence of skin manifestations was 29% (95% CI: 17.0–43.0), with a duration between 7 and 9 days, and most frequently affecting feet and hands (75%) and the trunk (71%). In general, the mean age was 44.3 ± 9.3 (17, 20–75), and the majority presented skin manifestations at the same time as other COVID-19 symptoms (43%) or more than 10 days after the onset of symptoms (34%), with itching (30%).

The skin is the main entry point for different microorganisms, and one of the main signalers of systemic infections through indirect manifestations (60, 61). Viral infections often manifest on the skin, such as measles (parvovirus B19), chickenpox, herpes zoster, dengue, and chikungunya (61–65). Therefore, health professionals may be careful to make the correct diagnosis based on skin manifestations.

The other reported symptoms were similar in frequency to skin manifestations: flulike symptoms (myalgia, headache, and/or asthenia in 41%, fever in 37%, respiratory symptoms in 31%, cough in 27%, and anosmia and/or ageusia in 21%). The skin manifestations frequency analysis with 95% CI was performed considering all studies that reported sufficient data. The identification that the prevalence of skin manifestations of COVID-19 in patients over 19 years of age is similar to that of other symptoms may favor the inclusion of these manifestations in suspected symptoms of COVID-19. This can help identify SARS-CoV-2 infection, promote better management of COVID-19, and favor the control of viral spread (60, 66).

Considering that smell and taste disorders are prominent indicators of SARS-CoV-2 virus infection (67), the prevalence of skin lesion in our study (21%) resembles the prevalence found in other articles. A narrative review estimates that anosmia can vary from 30 to 60% depending on the geographic region (68). A living systematic review found a 26% prevalence of ageusia (12).

Although most studies were performed in Asia (42%), they had the lowest prevalence of skin manifestations of COVID-19 (9, 95% CI: 5.0–14.0). We believe that the estimates of the skin manifestations of COVID-19 on the African continent and North America are overestimated because we had only one observational study conducted on these continents. Africa was the continent with the lowest number of studies (only 1 study) (48) and had the highest prevalence of skin manifestations of COVID-19 (61%). However, the sample in this study was small (*n* = 38), the confidence interval ranged from 44 to 76%, and the sample was not included in the confidence interval for general skin manifestations.

We observed great heterogeneity between studies by continent (I^2^ > 96% and *p* = 0), which was already expected considering the population variation in each geographic region. Other studies that evaluated the frequency of COVID-19 symptoms by geographic region found that the most prevalent symptoms may vary in different countries (56, 69).

In an analysis of skin manifestations by presentation subtype, we found that inflammatory lesions were the most prevalent (63%), with the majority presenting a maculopapular rash morphology. The inflammatory skin manifestations of viral infections may result from an immunological reaction of circulating antibodies and activated lymphocytes to combat the virus (70). Furthermore, some types of rash occur due to viral replication in epidermis and dermis cells and degranulation of mast cells (65, 71). This may explain the higher prevalence of rash in SARS-CoV-2 infection. Novak et al. (72) reported that rashes (mainly maculopapular, urticarial, and vesicular) were more frequent on the trunk, arms, and legs, which agrees with our findings.

Skin manifestations of vascular origin were the second most prevalent type, occurring in 9% of patients. The majority (66%) presented with livedoid lesions, purpura, and/or necrosis (95% CI: 33.0–93.0). Vascular skin manifestations appeared more frequently at the same time with other symptoms in the genital regions, arms, legs, hands, and feet and without other associated skin symptoms. The mean age of the patients was 67.2 ± 6.3 years, demonstrating that the patients were older. We consider that our estimate of vascular injuries may have been lower than the actual estimate, due to the exclusion in this review of studies that only reported severe vaso-occlusive injuries resulting from thrombosis.

There are reports that vaso-occlusive lesions may be associated with activation of the complement system resulting from the invasion of SARS-CoV-2 (71, 73, 74). This causes microvascular lesions in the endothelium and consequent vasculitis in different organs, including the skin (71, 73, 74). These lesions can manifest as livedo, purpura, necrosis, ischemia, and ecchymosis. Although we did not have enough data to perform an association analysis between age and the types of skin manifestations, Casas et al. (25) and a meta-analysis published by Jamshidi et al. (75) found that skin manifestations of vascular origin affected elderly people more than younger people. This may be due to greater exposure to medications, dysregulation of the immune system, and greater prevalence of comorbidities, among other hypotheses that suggest sensitization of the vascular endothelium (71). Vascular lesions can affect all parts of the body, including the trunk, genitals, and extremities (17, 72).

Chilblain-like and pernio-like lesions were the least frequent skin manifestations (5 and 2%, respectively). They appeared on the hands and feet, as reported in the literature (72). Pernio-like lesions also had associated pruritus in 22% of cases. Both lesions appeared more frequently 10 days after the onset of other COVID-19 symptoms. Patients with chilblain-like lesions were younger (mean age 30.4 ± 10.2), which agrees with the results of the study by Giavedoni et al. (30) and Landa et al. (76). Both lesions most frequently affect children, adolescents, and young adults (up to 30 years of age) (77–80).

Regarding the gender of patients with COVID-19, most studies did not report this information (48%). Among those who reported, the proportion of males and females was similar (27 and 25%, respectively). Female patients had a higher proportion of skin manifestations than male patients (20% vs. 13%). However, the meta-analysis showed that this association was not statistically significant (OR = 1.19; 95% CI: 0.8–1.8, I^2^: 28%, *p* = 0.44), with very low certainty of evidence.

The majority of patients included had mild/moderate COVID-19, without the need for oxygen support according to WHO criteria (55). The frequency of skin manifestations was similar between patients with severe/critical COVID-19 (23%) and those with mild/moderate COVID-19 (20%), with OR = 2.32, but was not statistically significant (95% CI: 0.8–7.0, I^2^: 82%, *p* = 0.14) with very low certainty of evidence. Sundus et al. (41) and Tan et al. (81) also found that patients with severe COVID-19 had more skin manifestations (*p* < 0.001).

There is still no consensus in the literature on the possible relationship between the severity of COVID-19 and skin manifestations. Studies hypothesize that it may be a drug reaction due to greater administration of drugs in severe or critical patients (25), or it may be due to a reaction caused by the cytokine storm produced by the immune system (82).

In another subgroup analysis by type of skin manifestations, we found that patients with severe/critical COVID-19 developed more skin manifestations of vascular origin (*p* = 0), while patients with mild/moderate COVID-19 presented more chilblain-like and pernio-like (p = 0) and inflammatory lesions (*p* > 0.05). Freeman et al. (29) and Sánchez-Cárdenas et al. (38) also found a significant association (*p* < 0.05) between vaso-occlusive lesions and severity of COVID-19. This may be due to microvascular lesions in the skin, which clinically manifest as vaso-occlusive lesions and are present in patients with severe COVID-19 infection (73, 74). Skin manifestations resulting from severe vascular injuries were an exclusion criterion for this systematic review. This may have limited our estimate of the frequency of skin manifestations of vascular origin. However, our results for vascular manifestations of the skin are consistent with what has been reported in the literature to date.

In this systematic review, we found that testing for SARS-CoV-2 was performed on skin biopsy samples from only 4 patients (28). Although all of them were negative, we consider that testing for COVID-19 in skin samples can help in diagnosing the disease and understanding the pathophysiological mechanisms of skin involvement by the SARS-CoV-2 virus. Studies suggest that testing skin samples can be an additional diagnostic method and help identify cases in which the PCR swab sample was not collected properly (83) and in cases in which patients did not acquire humoral immunity for serological testing (84). This can help reduce false-negative cases.

The advent of vaccines for COVID-19 and population adherence allowed control of COVID-19 (9, 85). The WHO declared the end of the COVID-19 public health emergency in May 2023 (85). COVID-19 variants emerged as a way for the virus to adapt and maintain its infectivity in different regions of the world (86–88). In January 2022, the largest viral circulation of SARS-CoV-2 was the Omicron variant (88). We intended to perform a meta-analysis by the period of the year, considering the pre- and post-vaccine period and the period of the highest incidence of COVID-19 variants (pre and post-omicron variant). However, it was not possible to analyze the association between the period of diagnosis (year) of COVID-19 and the frequency of skin manifestations because the studies presented data from January 2020 to August 2021 and did not individualize data by period of diagnosis. Only two studies presented data collected in 2021 (49, 52) All other studies presented data from only 2020 or 2020–2021.

We encourage further observational studies to evaluate the prevalence of cutaneous manifestations in all geographic regions, mainly on the African and North American continents. We also suggest that future research evaluate skin manifestations presented in different periods and with subgroups so that it is possible to evaluate the impact of vaccines and SARS-CoV-2 variants on the prevalence of skin manifestations of COVID-19. Furthermore, we suggest that skin biopsies be performed to aid in the diagnosis and understanding of the pathophysiological mechanisms of COVID-19 skin manifestations.

We propose that skin manifestations without a clear origin should be considered in the suspected diagnosis of COVID-19, given their prevalence, which is comparable to other symptoms in adults and the elderly.

Although our review included studies from several countries, we believe that the estimates of skin manifestations of COVID-19 on the African continent and North America are overestimated because we only had one observational study conducted on these continents. Another limitation is that no study presented data on skin manifestations per data collection period. This made it impossible to analyze the pre- and post-vaccine periods and periods of greater circulation of specific variants of the SARS-CoV-2 virus. We also added as exclusion criteria for this systematic review studies that only reported skin manifestations resulting from severe vascular injuries. This may have limited our estimate of the frequency of skin manifestations of vascular origin. However, our results for vascular manifestations of the skin are consistent with what has been reported in the literature to date.

There is an urgent need to identify cases of COVID-19 that may present with different extrapulmonary symptomatology. We demonstrated that skin manifestations have a similar prevalence to the main symptoms of COVID-19, such as fever, cough, anosmia, and ageusia. We also identified the geographic regions with the highest prevalence of skin manifestations of COVID-19. Our review provides data that can help healthcare professionals identify suspect cases of COVID-19 by evaluating skin manifestations and their morphological characteristics.

## 5 Conclusion

Among 10,121 COVID-19 positive patients, 29% showed skin manifestations. The highest prevalence was in Africa (61%). The subtypes of skin manifestations found were inflammatory manifestations (most with maculopapular rash), vascular (most with livedo/purpura/necrosis lesion), chilblain-like lesions, and pernio-like lesions. The trunk of the body was most affected by inflammatory lesions, arms and legs were affected by inflammatory and vascular lesions, and feet and hands were affected by chilblain-like and pernio-like lesions. We found no association between female/male and the severity of COVID-19 with general skin manifestations. However, manifestations of vascular origin were found only in elderly patients and were more frequent in patients with severe/critical COVID-19, with a statistically significant association with severity. Other lesions were found in younger patients and were more frequent in patients with mild/moderate COVID-19. We suggest that cutaneous manifestations without a clear origin should be considered in the suspected diagnosis of COVID-19 since the general prevalence is similar to the prevalence of other symptoms in adults and the elderly.

## Data Availability

The original contributions presented in the study are included in the article/Supplementary material, further inquiries can be directed to the corresponding author.
