# Supplementary material for: Prevalence of skin manifestations in patients with COVID-19: a systematic review and meta-analysis
Source: Front Med (Lausanne). 2024 Aug 21;11:1390775. doi: 10.3389/fmed.2024.1390775 (PMC11371801; doi:10.3389/fmed.2024.1390775)
Supplement: Supplementary file 1 [file Data_Sheet_1.docx]

Supplementary Material

**Prevalence of skin manifestations in patients with COVID-19: A systematic review and meta-analysis**

Beatriz Regina Lima de Aguiar, Elaine Barros Ferreira, Graziela De Luca Canto, Eliete Neves Silva Guerra, Paula Elaine Diniz dos Reis^*^

*** Correspondence:** Paula Elaine Diniz dos Reis, [pauladiniz@unb.br](mailto:pauladiniz@unb.br)

# Supplementary Figures and Tables

## Supplementary Tables

**Supplementary Table 1.** Search strategy performed in databases CINAHL, EMBASE, LILACS, LIVIVO, PubMed, Scopus, Web of Science Core Collection, Google Scholar, and ProQuest Dissertations & Thesis Global on June 06th, 2023.

| **Electronic Database** | **Search Strategy** | **Results** |
| --- | --- | --- |
| **CINAHL** | TX ( “acral lesions” OR “acral skin lesions” OR “acral purpuric lesions” OR “acro-ischemic” OR “acro-ischemia” OR “acrodermatitis” OR “chilblains” OR “chilblain” OR “chilblain-like” OR “chickenpox-like” OR “chicken-pox-like” OR “cutaneous lesions” OR “cutaneous manifestations” OR “cutaneous rashes” OR “cutaneous symptoms” OR “cutaneous vasculitis” OR “dermatitis” OR “dermatological manifestations” OR “eczema” OR “erythema multiforme” OR “erythematous maculo-papular” OR “erythematous papules” OR “erythematous rash” OR “erythemato-violaceous” OR “exanthema” OR “exanthems” OR “exanthem” OR “exanthematous” OR “livedo” OR “livedo” OR “livedoid” OR “maculopapular” OR “maculo-papular rash” OR “morbilliform rash” OR “papule” OR “papules” OR “papulous” OR “papulovesicular” OR “Petechiae” OR “pseudo-vesicles” OR “purpura” OR “purpuric” OR “rash” OR “skin” OR “Spongiosis” OR “targetoid lesions” OR “urticaria” OR “urticarial” OR “varicella-like” OR “varicelliform” OR “vascular skin lesions” OR “skin manifestations” OR “skin manifestation” OR “varicella-like exanthem” OR “vasculitic lesions” OR “vasculitic skin lesions” OR “vasculopathic lesions” OR “vasculopathic skin lesions” OR “vasculitic pattern” OR “vesicles” OR “vesicular eruptions” ) AND TX ( "2019 novel coronavirus" OR "covid19" OR "new coronavirus" OR "novel coronavirus" OR "COVID" OR "COVID-19" OR "COVID-2019" OR "severe acute respiratory syndrome coronavirus 2" OR "2019-nCoV" OR "SARS-CoV-2" OR "2019nCoV" ) | 3556 |
| **EMBASE** | ('acral lesions' OR 'acral skin lesions' OR 'acral purpuric lesions' OR 'acro-ischemic' OR 'acro-ischemia' OR 'acrodermatitis' OR 'chilblains' OR 'chilblain' OR 'chilblain-like' OR 'chickenpox-like' OR 'chicken-pox-like' OR 'cutaneous lesions' OR 'cutaneous manifestations' OR 'cutaneous rashes' OR 'cutaneous symptoms' OR 'cutaneous vasculitis' OR 'dermatitis' OR 'dermatological manifestations' OR 'eczema' OR 'erythema multiforme' OR 'erythematous maculo-papular' OR 'erythematous papules' OR 'erythematous rash' OR 'erythemato-violaceous' OR 'exanthema' OR 'exanthems' OR 'exanthem' OR 'exanthematous' OR 'livedo' OR 'livedoid' OR 'maculopapular' OR 'maculo-papular rash' OR 'morbilliform rash' OR 'papule' OR 'papules' OR 'papulous' OR 'papulovesicular' OR 'petechiae' OR 'pseudo-vesicles' OR 'purpura' OR 'purpuric' OR 'rash' OR 'skin' OR 'spongiosis' OR 'targetoid lesions' OR 'urticaria' OR 'urticarial' OR 'varicella-like' OR 'varicelliform' OR 'vascular skin lesions' OR 'skin manifestations' OR 'skin manifestation' OR 'varicella-like exanthem' OR 'vasculitic lesions' OR 'vasculitic skin lesions' OR 'vasculopathic lesions' OR 'vasculopathic skin lesions' OR 'vasculitic pattern' OR 'vesicles' OR 'vesicular eruptions') AND ('2019 novel coronavirus' OR 'covid19' OR 'new coronavirus' OR 'novel coronavirus' OR 'covid' OR 'covid-19' OR 'covid-2019' OR 'severe acute respiratory syndrome coronavirus 2' OR '2019-ncov' OR 'sars-cov-2' OR '2019ncov') AND [embase]/lim NOT ([embase]/lim AND [medline]/lim) AND ('article'/it OR 'article in press'/it) | 2473 |
| **LILACS** | (“SARS-CoV-2” OR “COVID-19” OR “Coronavirus”) AND (“Manifestações Cutâneas” OR “Skin Manifestations” OR “Manifestaciones Cutáneas” OR “Manifestations cutanées” OR “Exantema” OR “Exanthema” OR “Exanthème”) | 250 |
| **LIVIVO** | (“SARS-CoV-2” OR “COVID-19” OR “Coronavirus”) AND (“Skin Manifestations” OR “Exanthema”) | 1175 |
| **PubMed** | ("acral lesions"[All Fields] OR "acral skin lesions"[All Fields] OR "acral purpuric lesions"[All Fields] OR "acro-ischemic"[All Fields] OR "acro-ischemia"[All Fields] OR "acrodermatitis"[MeSH Terms] OR "acrodermatitis"[All Fields] OR "chilblains"[MeSH Terms] OR "chilblains"[All Fields] OR "chilblain"[All Fields] OR "chilblain-like"[All Fields] OR "chickenpox-like"[All Fields] OR "cutaneous lesions"[All Fields] OR "cutaneous manifestations"[All Fields] OR "cutaneous rashes"[All Fields] OR "cutaneous symptoms"[All Fields] OR "cutaneous vasculitis"[All Fields] OR "dermatitis"[MeSH Terms] OR "dermatitis"[All Fields] OR "dermatological manifestations"[All Fields] OR "eczema"[MeSH Terms] OR "eczema"[All Fields] OR "erythema multiforme"[MeSH Terms] OR "erythema multiforme"[All Fields] OR "erythematous maculo-papular"[All Fields] OR "erythematous papules"[All Fields] OR "erythematous rash"[All Fields] OR "erythemato-violaceous"[All Fields] OR "exanthema"[MeSH Terms] OR "exanthema"[All Fields] OR "exanthems"[All Fields] OR "exanthem"[All Fields] OR "exanthematous"[All Fields] OR "livedo"[All Fields] OR "livedo"[All Fields] OR "livedoid"[All Fields] OR "maculopapular"[All Fields] OR "maculo-papular rash"[All Fields] OR "morbilliform rash"[All Fields] OR "papule"[All Fields] OR "papules"[All Fields] OR "papulous"[All Fields] OR "papulovesicular"[All Fields] OR "Petechiae"[All Fields] OR "pseudo-vesicles"[All Fields] OR "purpura"[MeSH Terms] OR "purpura"[All Fields] OR "purpuric"[All Fields] OR "rash"[All Fields] OR "skin"[MeSH Terms] OR "skin"[All Fields] OR "Spongiosis"[All Fields] OR "targetoid lesions"[All Fields] OR "urticaria"[MeSH Terms] OR "urticaria"[All Fields] OR "urticarial"[All Fields] OR "varicella-like"[All Fields] OR "varicelliform"[All Fields] OR "vascular skin lesions"[All Fields] OR "skin manifestations"[MeSH Terms] OR "skin manifestations"[All Fields] OR "skin manifestation"[All Fields] OR "varicella-like exanthem"[All Fields] OR "vasculitic lesions"[All Fields] OR "vasculitic skin lesions"[All Fields] OR "vasculopathic lesions"[All Fields] OR "vasculopathic skin lesions"[All Fields] OR "vasculitic pattern"[All Fields] OR "vesicles"[All Fields] OR "vesicular eruptions"[All Fields]) AND ("2019 novel coronavirus"[All Fields] OR "covid19"[All Fields] OR "new coronavirus"[All Fields] OR "novel coronavirus"[All Fields] OR "COVID"[All Fields] OR "COVID-19"[MeSH Terms] OR "COVID-2019"[All Fields] OR "severe acute respiratory syndrome coronavirus 2"[All Fields] OR "2019-nCoV"[All Fields] OR "SARS-CoV-2"[MeSH Terms] OR "SARS-CoV-2"[All Fields] OR "2019nCoV"[All Fields]) | 7175 |
| **Scopus** | (TITLE-ABS-KEY ( "SARS-CoV-2" OR "COVID-19" OR "Coronavirus" ) AND TITLE-ABS-KEY ( "Skin Manifestations" OR "Exanthema" ) ) | 1373 |
| **Web of Science Core Collection** | (TS=(“acral lesions” OR “acral skin lesions” OR “acral purpuric lesions” OR “acro-ischemic” OR “acro-ischemia” OR “acrodermatitis” OR “chilblains” OR “chilblain” OR “chilblain-like” OR “chickenpox-like” OR “chicken-pox-like” OR “cutaneous lesions” OR “cutaneous manifestations” OR “cutaneous rashes” OR “cutaneous symptoms” OR “cutaneous vasculitis” OR “dermatitis” OR “dermatological manifestations” OR “eczema” OR “erythema multiforme” OR “erythematous maculo-papular” OR “erythematous papules” OR “erythematous rash” OR “erythemato-violaceous” OR “exanthema” OR “exanthems” OR “exanthem” OR “exanthematous” OR “livedo” OR “livedo” OR “livedoid” OR “maculopapular” OR “maculo-papular rash” OR “morbilliform rash” OR “papule” OR “papules” OR “populous” OR “papulovesicular” OR “petechiae” OR “pseudo-vesicles” OR “purpura” OR “purpuric” OR “rash” OR “skin” OR “Spongiosis” OR “targetoid lesions” OR “urticaria” OR “urticarial” OR “varicella-like” OR “varicelliform” OR “vascular skin lesions” OR “skin manifestations” OR “skin manifestation” OR “varicella-like exanthem” OR “vasculitic lesions” OR “vasculitic skin lesions” OR “vasculopathic lesions” OR “vasculopathic skin lesions” OR “vasculitic pattern” OR “vesicles” OR “vesicular eruptions”)) AND TS=("2019 novel coronavirus" OR "covid19" OR "new coronavirus" OR "novel coronavirus" OR "COVID" OR "COVID-19" OR "COVID-2019" OR "severe acute respiratory syndrome coronavirus 2" OR "2019-nCoV" OR "SARS-CoV-2" OR "2019ncovr") | 5944 |
| **Google Scholar** | (“SARS-CoV-2” OR “COVID-19” OR “Coronavirus”) AND (“Skin Manifestations” OR “Exanthema”) | 100 |
| **ProQuest Dissertations & Thesis Global** | (“SARS-CoV-2” OR “COVID-19” OR “Coronavirus”) AND (“Skin Manifestations” OR “Exanthema”) | 221 |

**Supplementary Table 2.** Excluded articles and reasons for exclusion (n= 86).

| **Reference** | **Reason for exclusion** |
| --- | --- |
| Alonso MN, Mata-Forte T, García-León N, et al. Incidence, Characteristics, Laboratory Findings and Outcomes in Acro-Ischemia in COVID-19 Patients. Vasc Health Risk Manag 2020; 16: 467-78. | 6 |
| Brazzelli V, Vassallo C, Barruscotti S, et al. Late-onset cutaneous eruption in hospitalized COVID-19 patients. Eur J Dermatol 2020; 30: 621-2. | 11 |
| Ciechanowicz P, Lewandowski K, Szymańska E, Kaniewska M, Rydzewska GM, Walecka I. Skin and gastrointestinal symptoms in COVID-19. Prz Gastroenterol 2020; 15: 301-8. | 10 |
| De Masson A, Bouaziz JD, Sulimovic L, et al. Chilblains is a common cutaneous finding during the COVID-19 pandemic: A retrospective nationwide study from France. J Am Acad Dermatol 2020; 83: 667-70. | 7 |
| Droesch C, Do MH, DeSancho M, Lee EJ, Magro C, Harp J. Livedoid and Purpuric Skin Eruptions Associated With Coagulopathy in Severe COVID-19. JAMA Dermatol 2020; 156: 1022–4. | 11 |
| Fernandez-Nieto D, Jimenez-Cauhe J, Suarez-Valle A, et al. Characterization of acute acral skin lesions in nonhospitalized patients: A case series of 132 patients during the COVID-19 outbreak. J Am Acad Dermatol 2020; 83: e61-e3. | 7 |
| Freeman EE, McMahon DE, Lipoff JB, et al. American Academy of Dermatology Ad Hoc Task Force on COVID-19. Pernio-like skin lesions associated with COVID-19: A case series of 318 patients from 8 countries. J Am Acad Dermatol 2020; 83: 486-92. | 7 |
| Gianotti R, Coggi A, Boggio F, Fellegara G. Similarities in Cutaneous Histopathological Patterns between COVID-19-positive and COVID-19 High-risk Patients with Skin Dermatosis. Acta Derm Venereol 2020; 100: adv00249. | 12 |
| Hubiche T, Cardot-Leccia N, Le Duff F, et al. Clinical, Laboratory, and Interferon-Alpha Response Characteristics of Patients With Chilblain-like Lesions During the COVID-19 Pandemic. JAMA Dermatol 2021; 157: 202-6. | 7 |
| Mascitti H, Bonsang B, Dinh A, et al. Clinical Cutaneous Features of Patients Infected With SARS-CoV-2 Hospitalized for Pneumonia: A Cross-sectional Study. Open Forum Infect Dis 2020; 7: ofaa394. | 4 |
| Piccolo V, Bassi A, Argenziano G, et al. Dermoscopy of chilblain-like lesions during the COVID-19 outbreak: A multicenter study on 10 patients. J Am Acad Dermatol 2020; 83: 1749-51. | 7 |
| Piccolo V, Neri I, Filippeschi C, et al. Chilblain-like lesions during COVID-19 epidemic: a preliminary study on 63 patients. J Eur Acad Dermatol Venereol 2020; 34: e291-3. | 1 |
| Rerknimitr P, Theerawattanawit C, Lertpichitkul P, et al. Skin manifestations in COVID-19: The tropics experience. J Dermatol 2020; 47: e444-6. | 7 |
| Rosell-Díaz AM, Mateos-Mayo A, Nieto-Benito LM, et al. Exanthema and eosinophilia in COVID-19 patients: has viral infection a role in drug induced exanthemas? J Eur Acad Dermatol Venereol. 2020; 34: e561-3. | 11 |
| Zhang JJ, Dong X, Cao YY, et al. Clinical characteristics of 140 patients infected with SARS-CoV-2 in Wuhan, China. Allergy 2020; 75: 1730-41. | 5 |
| Akca HM, Tuncer Kara K. Evaluation of urticaria patients before and during the period of the COVID-19 pandemic: A retrospective study. Dermatol Ther 2021; 34: e14800. | 2 |
| Al-Harbawi AL, Chalabi Q S, Alsalman H, Saeed M. Dermatological Manifestation in Coronavirus Disease 2019 Patients in Iraq. Macedonian J Med Scienc 2021; 9: 1085-91. | 7 |
| Al-Harbawi AL, Alsalman H, Chalabi Q S, Saeed M. Hair, nail and skin changes during COVID 19 era. J Pakist Assoc Dermatol 2021; 31: 441-6. | 7 |
| Ali A, Al-Shidhani S, Al-Balushi F, et al. Cutaneous Manifestations of COVID-19: An Experience From Oman. Cureus 2021; 13: e16667. | 7 |
| Almeida G, Arruda S, Marques E, Michalany N, Sadick N. Presentation and Management of Cutaneous Manifestations of COVID-19. J Drugs Dermatol 2021; 20: 76-83 | 7 |
| Anaba EL. Prevalence and pattern of acute dermatological manifestations of the novel COVID-19 infection at a COVID-19 treatment center in Sub-Saharan Africa. Dermatol Ther 2021; 34: e14953. | 7 |
| Birlutiu V, Feiereisz AI, Oprinca G, et al. Cutaneous manifestations associated with anosmia, ageusia and enteritis in SARS-CoV-2 infection - A possible pattern? Observational study and review of the literature. Int J Infect Dis 2021; 107: 72-7. | 12 |
| Böncüoğlu E, Kıymet E, Şahinkaya Ş, et al. Mucocutaneous Findings of Multisystem Inflammatory Syndrome in Children: A Single-Center Experience. J Trop Pediatr 2021; 67:fmab070. | 11 |
| Brancaccio G, Gussetti N, Sasset L, et al. Cutaneous manifestations in a series of 417 patients with SARS-CoV-2 infection: epidemiological and clinical correlates of chilblain like lesions. Pathog Glob Health 2021; 115:483-6. | 12 |
| Chowdhury ATM, Karim MR, Mehedi HH, et al. Analysis of the primary presenting symptoms and hematological findings of COVID-19 patients in Bangladesh. J Infect Dev Ctries 2021; 15: 214-23. | 7 |
| Dezoteux F, Mille B, Fievet C, et al. Vascular skin manifestations in patients with severe COVID-19 in intensive care units: a monocentric prospective study. Eur J Dermatol 2021; 31: 508–13. | 6 |
| Feito-Rodríguez M, Mayor-Ibarguren A, Cámara-Hijón C, et al. Chilblain-like lesions and COVID-19 infection: A prospective observational study at Spain's ground zero. J Am Acad Dermatol 2021; 84: 507-9. | 4 |
| Frumholtz L, Bouaziz JD, Battistella M, et al. Saint-Louis CORE (COvid REsearch). Type I interferon response and vascular alteration in chilblain-like lesions during the COVID-19 outbreak. Br J Dermatol 2021; 185: 1176-85. | 4 |
| Gannon R, Anne Fowles J, Gerrard C, Scott B. Prevalence of skin injuries in COVID-19 patients in a specialist UK respiratory Intensive Care Unit. Wounds UK 2021; 17: 1-4. | 5 |
| García-Gil MF, Monte-Serrano J, Lapeña-Casado A, et al. No antibody response in cutaneous manifestations associated with COVID-19: An observational study of 64 cases with microbiological and clinical characterization. Dermatol Ther 2021; 34: e15142. | 4 |
| Gimeno-Miguel A, Bliek-Bueno K, Poblador-Plou B, et al. PRECOVID Group. Chronic diseases associated with increased likelihood of hospitalization and mortality in 68,913 COVID-19 confirmed cases in Spain: A population-based cohort study. PLoS One 2021; 16: e0259822. | 5 |
| Hubiche T, Le Duff F, Fontas E, Rapp J, Chiaverini C, Passeron T. Relapse of chilblain-like lesions during the second wave of the COVID-19 pandemic: a cohort follow-up. Br J Dermatol 2021; 185: 858-9. | 7 |
| Kawen A, Saadoon A. COVID 19 patients/southern of Iraq: Character and dermatologic manifestations. Indian J Forensic Med Toxicol 2021; 15: 1624-33. | 12 |
| Kutlu Ö, Öğüt ND, Erbağcı E, Metin A. Dermatologic comorbidities of the patients with severe COVID-19: A case-control study. Dermatol Ther 2021; 34: e14731. | 2 |
| Marzano AV, Genovese G, Moltrasio C, et al. Italian Skin COVID-19 Network of the Italian Society of Dermatology and Sexually Transmitted Diseases. The clinical spectrum of COVID-19-associated cutaneous manifestations: An Italian multicenter study of 200 adult patients. J Am Acad Dermatol 2021; 84: 1356-63. | 4 |
| Mascitti H, Jourdain P, Bleibtreu A, et al. AP-HP/Universities/INSERM COVID-19 research collaboration. Prognosis of rash and chilblain-like lesions among outpatients with COVID-19: a large cohort study. Eur J Clin Microbiol Infect Dis 2021; 40: 2243-48. | 4 |
| McCleskey PE, Zimmerman B, Lieberman A, et al. Epidemiologic Analysis of Chilblains Cohorts Before and During the COVID-19 Pandemic. JAMA Dermatol 2021; 157: 947-53. | 4 |
| Miot HA, Ianhez M, Müller Ramos P. Self-reported cutaneous manifestations in 1429 Brazilian COVID-19-infected patients. J Eur Acad Dermatol Venereol 2021; 35: e172-3. | 12 |
| Mohta A, Mohta A, Nai RS, et al. An Observational Study of Mucocutaneous Manifestations among SARS-CoV-2 Patients from Three COVID-19 Dedicated Tertiary Care Centers. Indian Dermatol Online J 2021; 12: 687-95. | 12 |
| Muhammad A, Iftikhar N, Mashhood A, et al. Dermatological Manifestations of COVID-19 in Patients Reporting to a Tertiary Care Hospital in Rawalpindi, Pakistan. Cureus 2021; 13: e18973. | 12 |
| Nuno-Gonzalez A, Martin-Carrillo P, Magaletsky K, et al. Prevalence of mucocutaneous manifestations in 666 patients with COVID-19 in a field hospital in Spain: oral and palmoplantar findings. Br J Dermatol 2021; 184: 184-5. | 4 |
| Ortega-Quijano D, Fernandez-Nieto D, Jimenez-Cauhe J, Cortes-Cuevas JL, Marcos-Mencia D, Rodriguez-Dominguez M. Association between COVID-19 and chilblains: a case-control study. J Eur Acad Dermatol Venereol 2021; 35: e359-e61. | 7 |
| Otrofanowei E, Akinkugbe AO, Otike-Odibi BI, et al. Covid 19: A Prospective Observational Study on the Cutaneous Manifestations of Patients in Lagos, Nigeria. West Afr J Med 2021; 38: 944-51. | 7 |
| Pangti R, Gupta S, Nischal N, Trikha A. Recognizable vascular skin manifestations of SARS-CoV-2 (COVID-19) infection are uncommon in patients with darker skin phototypes. Clin Exp Dermatol 2021; 46: 180-2. | 12 |
| Poizeau F, Barbarot S, Le Corre Y, et al. Long-term Outcome of Chilblains Associated with SARS-CoV-2. Acta Derm Venereol 2021; 101: adv00614. | 12 |
| Punyaratabandhu P, Chirachanakul P. Cutaneous eruption in COVID-19-infected patients in Thailand: An observational descriptive study. J Dermatol 2021; 48: 14-20. | 11 |
| Rakita U, Kaundinya T, Guraya A, et al. Associations between onychomycosis and COVID-19 clinical outcomes: a retrospective cohort study from a US metropolitan center. Arch Dermatol Res 2022; 314: 897-902. | 9 |
| Rousselin A. Dermatological manifestations and its association with SARS-CoV-2: a descriptive cross-sectional study from Guatemala. An Bras Dermatol 2021; 96: 106-7. | 7 |
| Rrapi R, Chand S, Lo JA, et al. The significance of exanthems in COVID-19 patients hospitalized at a tertiary care centre. J Eur Acad Dermatol Venereol 2021; 35: e640-e2. | 10 |
| Singh GK, Mitra B, Bhatnagar A, et al. Unusual Spurts of Rosacea Like Dermatoses, Posing a Diagnostic Dilemma During Covid-19 Pandemic: A Cross-Sectional, Observational Study From a Tertiary Care Centre. Indian J Dermatol 2021; 66: 401-4. | 3 |
| Unterluggauer L, Pospischil I, Krall C, et al. Cutaneous manifestations of SARS-CoV-2: A 2-center, prospective, case-controlled study. J Am Acad Dermatol 2021; 85: 202-4. | 12 |
| Visconti A, Bataille V, Rossi N, et al. Diagnostic value of cutaneous manifestation of SARS-CoV-2 infection. Br J Dermatol 2021; 184: 880-7. | 7 |
| Alimohamadi Y, Sepandi M, Rashti R, Sedighinezhad H, Afrashteh S. COVID-19: Clinical features, case fatality, and the effect of symptoms on mortality in hospitalized cases in Iran. J Taibah Univ Med Sci 2022; 17: 725-31. | 4 |
| Amendola A, Canuti M, Bianchi S, et al. Molecular evidence for SARS-CoV-2 in samples collected from patients with morbilliform eruptions since late 2019 in Lombardy, northern Italy. Environ Res 2022; 215: n113979. | 12 |
| Bdaiwi S, Abdul-Saheb R. Mucocutaneous Manifestations of COVID-19 among Iraqi Patients in Al Diwaniyah Province, Iraq: Case Series Study. J Communicable Diseases 2022; 39:244. | 4 |
| Bek LM, Berentschot JC, Heijenbrok-Kal MH, et al. Symptoms persisting after hospitalisation for COVID-19: 12 months interim results of the CO-FLOW study. ERJ Open Res 2022; 8: 00355-2022. | 14 |
| Bryan A, Samant H, Asarkar A, Nathan CO, Khandelwal A. Cutaneous Manifestations in COVID-19-Positive African American Patients. Ochsner J 2022; 22: 22-5. | 12 |
| Carmona RCC, Machado BC, Reis FC, et al. Hand, foot, and mouth disease outbreak by Coxsackievirus A6 during COVID-19 pandemic in 2021, São Paulo, Brazil. J Clin Virol 2022; 154: 105245. | 9 |
| Dear K, Psomadakis C, Dost S, et al. Cutaneous manifestations of paediatric multisystem inflammatory syndrome temporally associated with SARS-CoV-2: a single-centre experience. Br J Dermatol 2022; 186: 902-3. | 1 |
| Deo N, Tekin A, Bansal V, et al. From The Society of Critical Care Medicine Discovery Viral Infection and Respiratory Illness Universal Study (VIRUS): COVID-19 Registry Investigator Group. Cutaneous manifestations of hospitalized COVID-19 patients in the VIRUS COVID-19 registry. Int J Dermatol 2022; 61: 623-5. | 12 |
| Farag A, Labeeb A, Amin M, Elshaib M, Elnaidany, N. Dermatological Manifestations Associated with Covid-19 Patients in Elbagour General Hospital, El-Menoufia Governorate. Egypt J Hosp Med 2022; 88: 3916-24. | 7 |
| Gehlhausen JR, Little AJ, Ko CJ, et al. Lack of association between pandemic chilblains and SARS-CoV-2 infection. Proc Natl Acad Sci USA 2022; 119: e2122090119. | 11 |
| Lane JB, Stahly S, Sills A, et al. Examination of Cutaneous Changes Among Patients Following SARS-CoV-2 Infection. Cureus 2022; 14: e27052. | 11 |
| Lin J, Shen P, Zhang Y, et al. Epidemiology, diagnosis and treatment of early COVID-19 patients in Jiaxing City, Zhejiang Province, China, January to March 2020. Ann Palliat Med 2022; 11: 3472-82. | 10 |
| Mitamura Y, Schulz D, Oro S, et al. Cutaneous and systemic hyperinflammation drives maculopapular drug exanthema in severely ill COVID-19 patients. Allergy 2022; 77: 595-608. | 10 |
| Navarro-Bielsa A, Abadías-Granado I, Morales-Callaghan AM, et al. Experience with Cutaneous Manifestations in COVID-19 Patients during the Pandemic. J Clin Med 2022; 11: 600. | 7 |
| Poizeau F, Oger E, Barbarot S, et al. Chilblains during lockdown are associated with household exposure to SARS-CoV-2: a multicentre case-control study. Clin Microbiol Infect 2022; 28: 285-91. | 7 |
| Proietti I, Tolino E, Mambrin A, et al. Non-invasive instrumental examinations of cutaneous, adnexal and mucosal manifestations after SARS-COV-2 infection in adult and children. J Eur Acad Dermatol Venereol 2022; 36: e169-e70. | 6 |
| Roh DE, Lim YT, Kwon JE, Kim YH. Kawasaki disease following SARS-CoV-2 infection: Stronger inflammation with no increase in cardiac complications. Front Pediatr 2022; 10: 1036306. | 1 |
| Rybak-d'Obyrn J, Placek W, Owczarczyk-Saczonek A, et al. Cutaneous Eruptions in the Course of COVID-19 Among Geriatric Patients in a Nursing Home in Poland. Clin Cosmet Investig Dermatol 2022; 15: 2117-27. | 11 |
| Saberian P, Pazooki B, Hasani-Sharamin P, et al. Persistent/Late-Onset Complications of COVID-19 in General Population: A Cross-Sectional Study in Tehran, Iran. Int J Community Based Nurs Midwifery 2022; 10: 234-45. | 12 |
| Samannodi M, Alwafi H, Naser AY, et al. Determinants of Post-COVID-19 Conditions among SARS-CoV-2-Infected Patients in Saudi Arabia: A Web-Based Cross-Sectional Study. Diseases 2022;10: 55. | 7 |
| Sardana K, Sinha S, Yadav A, Deepak D, Panesar S, Mathachan SR. Paucity of cutaneous manifestations of COVID-19 among inpatients at a referral hospital in India. JAAD Int 2022; 8: 10-5. | 7 |
| Sawires R, Pearce C, Fahey M, Clothier H, Gardner K, Buttery J. Snotwatch COVID-toes: An ecological study of chilblains and COVID-19 diagnoses in Victoria, Australia. PLOS Glob Public Health 2022; 2: e0000488. | 12 |
| Singh V, Arya S, Shrivastva A, Paliwal A. Covid 19 and dermatological manifestations. Europ J of Mol Clin Med 2022; 9: 285-90. | 12 |
| Sugai T, Fujita Y, Inamura E, Maya Y, Shimizu S. Prevalence and patterns of cutaneous manifestations in 1245 COVID-19 patients in Japan: a single-centre study. J Eur Acad Dermatol Venereol 2022; 36: e522-e4. | 11 |
| Tamai M, Sakamoto R, Goto N, et al. Cutaneous manifestations of coronavirus disease 2019 patients in Japan. J Dermatol 2022;49: 872-8. | 12 |
| Tan CC, Dofitas BL, Frez MLF, Yap CDD, Uy JKK, Ciriaco-Tan CP. Cutaneous manifestations of COVID-19 in a tertiary COVID-19 referral hospital in the Philippines. JAAD Int 2022; 7: 44-51. | 7 |
| Visconti A, Murray B, Rossi N, et al. Cutaneous manifestations of SARS-CoV-2 infection during the Delta and Omicron waves in 348 691 UK users of the UK ZOE COVID Study app. Br J Dermatol 2022; 187: 900-8. | 7 |
| Alshiyab DM, Al-Qarqaz FA, Alhaje E, et al. Skin Manifestations Among Patients Admitted with COVID-19: A Cross-Sectional Study at a University-Based Tertiary Hospital in Jordan. Clin Cosmet Investig Dermatol 2023; 16: 1331-40. | 7 |
| Bougea A, Georgakopoulou VE, Palkopoulou M, et al. New‑onset non‑motor symptoms in patients with Parkinson's disease and post‑COVID‑19 syndrome: A prospective cross‑sectional study. Med Int (Lond) 2023; 3: 23. | 2 |
| Fatima A, Fasih F, Baig S, Khan F, Nseem S, Qureshi M. Seroprevalence of SARS-CoV-2 (COVID-19) antibodies in tertiary care hospital of Karachi, Pakistan. Rawal Med J 2023; 48: 70. | 4 |
| Kumar P, Radha G, Muthukrishnan M, Chandrasekaran B, Subbiah P, Raman J. Cutaneous Manifestations Associated with COVID-19 Infection in a COVID-Designated Hospital in North Chennai - A Descriptive Cross-Sectional Study. Indian Dermatol Online J 2022; 14: 67-71. | 12 |
| Mohta A, Pareek S, Sharma MK, et al. Hand Foot Mouth Disease During the SARS-CoV-2 Pandemic: A Multicentric Study. Indian Pediatr 2023; 60: 394-6. | 2 |
| Narang J, Gallop J, Cassard L, Seck S, Fernandez AP. Vascular cutaneous manifestations, especially in men, are associated with a more severe disease course in COVID-19 patients. Int J Dermatol 2023; 62: 271-4. | 12 |
| Skourtis A, Ekmektzoglou K, Xanthos T, Stouraitou S, Iacovidou N. Non-Typical Clinical Presentation of COVID-19 Patients in Association with Disease Severity and Length of Hospital Stay. J Pers Med 2023; 13: 132. | 7 |

**Reason for exclusion**

(1) Studies evaluating skin manifestations of COVID-19 in individuals under 19 years of age (children and adolescents) (n=3);

(2) Studies that do not report whether adult patients had a positive diagnosis, confirmed by Polymerase Chain Reaction (PCR) test, serology or antigen test, for COVID-19 (n=4);

(3) Studies in which adult patients had a negative PCR, serology or antigen test for COVID-19 (n=1);

(4) Studies that did not individualize data for adult patients with a confirmed diagnosis of COVID-19 by laboratory test (n=11);

(5) Patients with skin manifestations related to diseases other than COVID-19 (n=3);

(6) Patients with only skin manifestations of severe vasculopathies (vaso occlusive) (n=3);

(7) Studies that did not individualize results of skin manifestations of COVID-19 for adult or elderly patients (mixed samples) (n=26);

(8) Studies that reported skin manifestations associated with adverse vaccine reactions (n=0);

(9) Studies that reported skin manifestations associated with other infections, that not SARS-CoV-2 (n=2);

(10) Studies that reported skin manifestations associated with adverse drug reactions (n=4);

(11) Clinical trials, reviews, book chapters, letters, personal opinions, conference abstracts, case reports, and case series (n=9);

(12) Studies that did not report sufficient information (n=19);

(13) Studies in languages that do not use the latin-roman alphabet (n=0);

(14) Skin manifestations of COVID-19 after three months of diagnosis (n=1).

**Supplementary Table 3.** Characteristics of the included studies and main sample data (n=31).

| **Authors, year**  **Country** | **Type of study**  **Data collection period** | **Sample (n) /**  **Sex (n)** | **Mean age ± SD**  **(Range) in years** | **Covid-19 signs and symptoms (%)*** | **COVID-19 test** | **COVID-19 severity (%)** |
| --- | --- | --- | --- | --- | --- | --- |
| Askin et al (2020) (24)  Turkey | Prospective cohort  April 2020 | 210  M: 123  F: 87 | M: 57.44 ± 17.259  F: 58.80 ± 15.918 | NI | PCR | Wards (77.6)  ICU (22.4) |
| Casas et al (2020) (25)  Spain | Cross-sectional  April 3-16, 2020 | 375  M: 153  F: 222 | Pseudo-chilblain  32.5 ±21.8  Vesicular  45.6 ±20  Urticarial  48.7 ±19.9  Maculopapules  55.3 ±20. .2  Livedo/ necrosis  63.1 ±17.3 | Fever (74.7), cough (69.1), dyspnea (45. .6), asthenia (60.3), headache (33.9), nausea/vomiting/diarrhea (28.5), anosmia, ageusia (24.0), pneumonia (48.8) | NI | Hospitalization (47.2)  ICU or noninvasive mechanical ventilation (10.7) |
| Dalal et al (2020) (26)  India | Prospective cohort  for 3 weeks before June 2020 | 102  M: 95  F: 7 | 39.30 ± 17.9 | Symptomatic (26.5), asymptomatic (73.5) | PCR | Mild/moderate (26.5) |
| De Giorgi et al (2020) (27)  China and Italy | Cross-sectional  January 1 - March 15, 2020 | 678  M: 32**  F: 21** | 55.9**  (28-69) | NI | PCR | Mild (57.8)  Common (18.7)  Severe (17.5)  Critical (6.0) |
| Fernandez-Nieto et al (2020) (28)  Spain | Cross-sectional  March 1 – April 20, 2020 | 24  M: 6  F: 18 | Median 45.0  (19–65) | NI | PCR | Mild (58.3)  Pneumonia (41.7)  ICU (4.2) |
| Freeman et al (2020) (29)  International registry from 31 countries | Cross-sectional  April 8 – May 17, 2020 | 682  M: 329  F: 353 | Median 30  (19-49) | Fever (62.4), cough (55.8), shortness of breath (38.8), sore throat (37.6), headache (32.7), diarrhea/vomiting/nausea (30.9), malaise (27.3), myalgia (21.2), irritability/confusion (16.4), chest pain (15.2), abdominal pain (13.9), anosmia (10.9), dysgeusia (7.3), arthralgia (9.7), rhinorrhea (8.5), asymptomatic (6.7)** | PCR or Serology (IgG+IgM) | Oupatient care (66.0)**  Hospitalizated (34.0)** |
| Giavedoni et al (2020) (30)  Spain | Prospective cohort  April 1 – May 1, 2020 | 58  M: 31  F: 27 | Median 54.8 /  IQR 38.7-69 | Fever (67.2), cough (55.2), dyspnea (41.4), asthenia (27.6), myalgias (25.9), diarrhea (22.4), ageusia (17.2), anosmia (12.1), headache (6.9), vomiting (5.2) | PCR or  Serology  (IgG+IgM+ IgA) | Hospitalizated (65.5)  ICU (33.0)  Death (3.0) |
| Jasim et al (2020) (31)  Iraq | Retrospective cohort  March 1 – June 1, 2020 | 369  M: 140 F: 229 | 51 ± 14 | Symptomatic (67.0), asymptomatic (33.0)** | PCR | Hospitalizated (100)  Ward (80.5)  ICU (19.5)  Death (6.5) |
| Maestro et al (2020) (32)  Spain | Cross-sectional  April 14-30, 2020 | 75  M: 48  F: 27 | 67.5 ± 3.0 | NI | PCR or Serology (NI) | Mild/Moderate (100) |
| Carvalho-Schneider et al (2021) (33)  France | Prospective cohort  March 17 – June 3, 2020 | 150  M: 66  F: 84 | 49 ± 15 | Fever (51.4), dyspnea/shortness of breath (42.2), other respiratory signs (91.2), chest pain (14.0), abnormal auscultation (39.3), flulike symptoms (87.2), digestive disorders (33.1), anosmia/ageusia (59.3), diarrhea (30.8) | PCR | Mild/moderate (77.3)  Severe (22.7) |
| Dhamale et al (2021) (34)  India | Prospective cohort  NI | 303  M: 197  F: 106 | 40.25 ± 16.17 | Fever (45.8), chills (9.5), cough (41.9), sore throat (12.2), dyspnea (24.7), muscle pain (13.2), abdominal pain (3.3), diarrhea (4.9), nausea/vomiting (3.3), anosmia (0.3) | PCR | NI |
| Gianmarco et al (2021) (35)  Italy | Cross-sectional  March 2020 – January 2021 | 126  M: 41  F: 85 | 39  (22-67) | Fever (32.5), fatigue (49.2), anosmia (17.5), ageusia (14.0), diarrhea (11.9), dyspnea (7.9), dry and irritating cough (26.2) | nasopharyngeal swab | NI |
| Jacquin-Porretaz et al (2021) (36)  France | Prospective cohort  April 1 – July 1, 2020 | 30  M: 13  F: 17 | (20-95) | NI | PCR or  Serology  (IgG) | NI |
| Rekhtman et al (2021) (37)  United States | Prospective cohort  May 11 – June 15, 2020 | 296  Rash  M: 25  F: 10  No rash  M: 159  F: 102 | Median / IQR  Rash  64 /57-77  No rash  65 /55-74 | NI | PCR or Serology (IgG/IgM) | Invasive mechanical ventilation (31.4) |
| Sánchez Cárdenas et al (2021) (38)  Mexico | Prospective cohort  September 2020 – January 2021 | 210  M: 20**  F: 13** | 59.12 ± 13.14** | NI | PCR | Mild/Moderate (18.2)**  Severe (81.8)**  Death (63.6)** |
| Sharif et al (2021) (39)  Pakistan | Cross-sectional  March 2020 – December 2020 | 150  M: 94  F: 56 | 51.9 ± 17.2 | NI | PCR | NI |
| Solak et al (2021) (40)  Turkey | Cross-sectional  NI | 382  M: 195  F: 187 | Median 39 /  IQR 14-86 | Fever (31.9), nasal itching (11.5) | PCR | Mild/Moderate (96.6)  Severe/Critical (3.4) |
| Sundus et al (2021) (41)  Arab Emirates | Cross-sectional  May – July 2020 | 412  M: 370  F: 42 | 44 ± 16.32 | NI  Asymptomatic (46.8) | PCR | Mild (24.5)  Moderate (20.6)  Severe (8.0) |
| Thuangtong et al (2021) (42)  Thailand | Cross-sectional  January – September , 2020 | 93  M: 54  F: 39 | 40.8 ± 15.1  (21-83) | Symptoms of upper respiratory tract (71.0), lower respiratory tract (28.0), asymptomatic (1.0) | PCR | Mild (71.0)  Moderate (9.7)  Severe (14.0)  Critical (4.3) |
| Yildiray et al (2021) (43)  Turkey | Prospective cohort  March – June 2020 | 266  M: 92  F: 174 | 49.2 ± 18.69  (18–96) | Fever (80.0), myalgia (60.0), cough (60.0), fatigue (40.0) diarrhea (40.0), respiratory distress (20.0), sore throat (20.0)** | PCR | ICU (8.7)  Inpatient Clinic (53.0)  Quarantine Unit (38.3) |
| Dupont et al (2022) (44)  Brazil | Cross-sectional  March 17, 2020 – November 17, 2020 | 2,968  M: NI  F: NI | >18 y | NI | PCR | NI |
| Farhood et al (2022) (45)  Iraq | Cross-sectional  October 2020 – February 2021 | 100  M: 41  F: 59 | 34.25 ± 11.848  (19-62) | NI | PCR | Mild (51.0)  Moderate (45.0)  Severe (4.0) |
| Ghafoor et al (2022) (46)  Pakistan | Cross-sectional  July 31, 2020 – August 1, 2021 | 1026  M: NI  F: NI | 41.34 ± 12.08 | NI | PCR | NI |
| Mohammed et al (2022) (47)  Egypt and Saudi Arabia | Cross-sectional  December 2020 – July 2021 | 273  M: NI  F: NI | 36.5 ± 7.9 | Cough (64.5), diarrhea (56.0), fever (54.2), muscle pain (68.5), loss of smell (64.0), vomiting (35.0), loss of taste (33.5), sore throat (33.0), mild headache (24.0), fatigue/weakness (22.3) | PCR | Mild (53.1)  Moderate (39.2)  Severe (7.7) |
| Mostafa et al (2022) (48)  Egypt | Retrospective cohort  October – December 2020 | 38  M: 13  F: 25 | 41.2 ± 13.5  (20-70) | Fever (60.5), sore throat (28.9), cough (44.7), boneaches (10.5), dyspnea (13.2), flulike (5.3), runny nose (15.8), headache (21. .1), anosmia (10.5), respiratory distress (2.6), diarrhea (2.6), decreased O2 sat in attacks (5.3), asymptomatic (29.0), asymptomatic (23.7) | PCR | Mild (55.3)  Moderate (18.4)  Severe (2.6) |
| Niyatiwatchanchai et al (2022) (49)  Thailand | Cross-sectional  April – May, 2020 | 105  M: 54  F: 51 | Non-severe  30.8 ± 14.3  Severe  44.5 ± 14.5 | Fever (2.9), cough (20.0), dyspnea (9.5), wheeze (1.9), purulent sputum (1.9), chest pain (8.6), sore throat (5.7), rhinorrhea (9.5), headache (6.7), muscle pain (8.6), fatigue (12.4), nausea/vomiting (1.9), diarrhea (4.8), anosmia (1.9), ageusia (2.9), asymptomatic (52.4) | PCR | Non-severe (64.8)  Severe (35.2) |
| Pardal et al (2022) (50)  Argentina | Cross-sectional  May 1 – November 30, 2020 | 45  M: 31  F: 14 | 46 ± 17 | Fever (75.6), pneumonia (75.6), anosmia/ageusia (17.8), gastrointestinal symptoms (15.6), flu-like symptoms (17.8), thrombosis (2.2), asymptomatic (4.4) | PCR | Mild (15.6)  Moderate (44.4)    Severe (35.6)  ICU (24.4) |
| Parmar et al (2022) (51)  UK  England | Prospective cohort  March 1 – June 30 2020 | 93  M: 3**  F: 3** | (43-91) | Fever (66.7) dyspnea (66.7), cough (50.0)** | NI | Mild (50.0)**    Death (33.4)**    ICU (16.7)** |
| Tatliparmak et al (2022) (52)  Turkey | Prospective cohort  May - July 2021 | 192  M: 124  F: 68 | Median 45 /  IQR 33-54 | NI | PCR | Mild/Moderate (100) |
| Zengarini et al (2022) (53)  Italy | Cross-sectional  April 1, 2020 – April 30, 2021 | 1053  M: 421  F: 632 | 63.5 ± 17.322 | NI | PCR | NI |
| Cestari et al (2023) (54)  Brazil | Cross-sectional  February – June 2020 | 50  M: 27  F: 23 | 57 ± 18.6 | Fever (61.2), cough (63.3), myalgia (61.2), dyspnea (51.0), headache (28.6), anosmia (20.4) | PCR | Mild/Moderate (24.0)  Severe/Critical (76.0) |

Abbreviations - M: Male; F: Female; IQR: Interquartile Range; NI: No Informed; PCR: Polymerase Chain Reaction; IgG: Immunoglobulins G; IgM: Immunoglobulin M; IgA: Immunoglobulin A; ICU: Intensive Care Unit. ^a^ Except cutaneous manifestations; ^b^ The authors only presented the data of the patients who had a skin manifestation.

**Supplementary Table 4.** Critical appraisal checklist for included studies.

| **JBI Critical Appraisal Checklist for Prevalence Studies^a^** | | | | | | | | | |
| --- | --- | --- | --- | --- | --- | --- | --- | --- | --- |
| **Study** | **Q1** | **Q2** | **Q3** | **Q4** | **Q5** | **Q6** | **Q7** | **Q8** | **Q9** |
| Askin et al (2020) (24) | Y | U | U | N | Y | U | U | Y | Y |
| Casas et al (2020) (25) | Y | U | Y | N | Y | Y | Y | Y | Y |
| Dalal et al (2020) (26) | Y | N | U | Y | Y | Y | Y | Y | Y |
| De Giorgi et al (2020) (27) | Y | N | U | Y | Y | Y | Y | Y | Y |
| Fernandez-Nieto et al (2020) (28) | Y | N | U | Y | Y | Y | Y | Y | Y |
| Freeman et al (2020) (29) | Y | N | U | Y | Y | U | U | Y | Y |
| Giavedoni et al (2020) (30) | Y | U | U | N | Y | Y | Y | Y | Y |
| Jasim et al (2020) (31) | Y | U | U | Y | Y | U | U | Y | Y |
| Maestro et al (2020) (32) | Y | U | U | Y | Y | Y | Y | U | Y |
| Carvalho-Schneider et al (2021) (33) | Y | U | U | Y | Y | U | U | Y | Y |
| Dhamale et al (2021) (34) | Y | U | U | Y | Y | Y | Y | Y | Y |
| Gianmarco et al (2021) (35) | Y | U | U | Y | Y | U | U | Y | Y |
| Jacquin-Porretaz et al (2021) (36) | Y | Y | Y | Y | Y | U | U | Y | Y |
| Rekhtman et al (2021) (37) | Y | Y | Y | Y | Y | Y | Y | Y | Y |
| Sánchez Cárdenas et al (2021) (38) | Y | U | U | Y | Y | Y | Y | Y | Y |
| Sharif et al (2021) (39) | Y | N | U | Y | Y | Y | Y | Y | Y |
| Solak et al (2021) (40) | Y | U | U | Y | Y | U | U | U | Y |
| Sundus et al (2021) (41) | Y | N | Y | Y | Y | Y | Y | Y | Y |
| Thuangtong et al (2021) (42) | Y | U | U | Y | Y | U | U | Y | Y |
| Yildiray et al (2021) (43) | Y | U | U | Y | Y | Y | Y | Y | Y |
| Dupont et al (2022) (44) | Y | U | U | Y | Y | U | U | Y | Y |
| Farhood et al (2022) (45) | Y | U | U | Y | Y | Y | Y | Y | Y |
| Ghafoor et al (2022) (46) | Y | U | U | Y | Y | Y | Y | Y | Y |
| Mohammed et al (2022) (47) | Y | U | U | U | U | U | U | Y | Y |
| Mostafa et al (2022) (48) | Y | Y | Y | Y | Y | U | U | Y | Y |
| Niyatiwatchanchai et al (2022) (49) | Y | U | Y | Y | Y | Y | Y | Y | Y |
| Pardal et al (2022) (50) | Y | U | U | Y | Y | Y | Y | Y | Y |
| Parmar et al (2022) (51) | Y | U | U | Y | Y | Y | Y | Y | Y |
| Tatliparmak et al (2022) (52) | Y | N | U | Y | Y | Y | Y | Y | Y |
| Zengarini et al (2022) (53) | Y | U | U | Y | Y | Y | Y | U | Y |
| Cestari et al (2023) (54) | Y | U | U | Y | Y | Y | Y | Y | Y |
| Total (%) | 100 | 19 | 28 | 78 | 83 | 67 | 64 | 92 | 100 |

Abbreviations - Y, yes; No, no; U, unclear; N/A, not applicable. ^a^ Q1. Was the sample frame appropriate for addressing the target population? Q2. Were the study participants sampled in an appropriate way? Q3. Was the sample size adequate? Q4. Were the study subjects and the setting described in detail? Q5. Was the data analysis conducted with sufficient coverage of the identified sample? Q6. Were valid methods used to identify the condition? Q7. Was the condition measured in a standard, reliable way for all participants? Q8. Was there an appropriate statistical analysis? Q9. Was the response rate adequate, and if not, was the low response rate managed appropriately?

**Supplementary Table 5.** Frequency and morphological characteristics of cutaneous manifestations presented by positive and negative patients for COVID-19 in laboratory tests (n=31).

| **Authors, year** | **Total sample (n)** | **Confirmed COVID-19** | | **Non-Confirmed COVID-19** | | |
| --- | --- | --- | --- | --- | --- | --- |
|  |  | **Sample (n)** | **Skin manifestation (n)**  **Type (n)** | **Sample (n)** | | **Skin manifestation (n)**  **Type (n)** |
| Askin et al (2020) (24) | 210 | 122 | 34  NS | 88 | | 18  NS |
| Casas et al (2020) (25) | 375 | 234 | 234  pseudo-chilblain (29), vesicular (17), urticarial (49), maculopapules (122), livedo/necrosis(17) | 141 | | 141  pseudo-chilblain (42), vesicular (17), urticarial (24), maculopapules (54), livedo/necrosis (4) |
| Dalal et al (2020) (26) | 102 | 102 | 5  maculopapular (3), urticarial (2) | - | | - |
| De Giorgi et al (2020) (27) | 678 | 678 | 53  erythematous rash (macular, papular, maculopapular, and erythema multiforme-like eruptions) (37), diffuse urticaria (14), vesicular rash (2) | - | | - |
| Fernandez-Nieto et al (2020) (28) | 24 | 24 | 24  vesicular (24) | - | | - |
| Freeman et al (2020) (29) | 682 | 171 | 166  pernio (31), morbilliform (38), urticarial (27), macular erythema (23), vesicular (18), papulosquamous (17), livedo reticularis-like (9), erythroderma (4), grover-like (papulo-vesicular eruptions) (9), retiform purpura (11), petechial (5), erythema nodosum (2), acroischemia (7), bullous (3) | 511 | | 509  pernio (391), morbilliform (25), urticarial (28), macular erythema (29), vesicular (31), papulosquamous (15), livedo reticularis-like (15), erythroderma (17), grover-like (papulo-vesicular eruptions) (10), retiform purpura (7), petechial (11), pustular (5), erythema nodosum (3), acroischemia (41), bullous (11) |
| Giavedoni et al (2020) (30) | 58 | 41 | 31  chilblain-like (7), generalized maculo-papular (10), papulo-vesicular eruptions (6), urticarial (4), livedo reticularis (4) | 17 | | 14  chilblain-like (10), generalized maculo-papular (2), papulo-vesicular eruptions (2) |
| Jasim et al (2020) (31) | 369 | 369 | 18  maculopapular (8), petechial (1), pseudo-chilblain (2), pustular (3), urticarial (3), purpuric (1) | - | | - |
| Maestro et al (2020) (32) | 75 | 75 | 14  acral erythema-edema (6), maculopapular rash (4), urticarial (2), vesicular eruption (1), livedo reticularis-like lesions (1) | - | | - |
| Carvalho-Schneider et al (2021) (33) | 150 | 150 | 21 skin manifestation in d30  NS | - | | - |
| Dhamale et al (2021) (34) | 303 | 303 | 4  urticarial (3), maculopapular (1) | - | | - |
| Gianmarco et al (2021) (35) | 126 | 126 | 18  eczema (18) | - | | - |
| Jacquin-Porretaz et al (2021) (36) | 30 | 14 | 14  pseudo-chilblain (2), maculopapular rash (6), vesicular rash (2), purpuric (1), urticaria (3) | 16 | | 16  pseudo-chilblain (10), maculopapular rash (3), vesicular rash (3), eczema (2), purpuric rash (2) |
| Rekhtman et al (2021) (37) | 296 | 296 | 35  ulcer (13), purpura (9), necrosis (5), erythema (4), morbilliform rash (4), pernio-like (4), vesicular (1) | - | | - |
| Sánchez Cárdenas et al (2021) (38) | 210 | 210 | 32  vesicles (3), maculopapular (2), pernio (7), erythema (3) livedo (1), purpura (1), acroischemia (15) | - | | - |
| Sharif et al (2021) (39) | 150 | 110 | 7  bullous (1), urticarial lesions (1), vesicular eruption (1), cutaneous necrosis (1), maculopapular rash (2) or erythema elevatum dinutum like (1) | 40 | | 0 |
| Solak et al (2021) (40) | 382 | 382 | 70  Rash (69): not especified (33), erythema (29), and wounds (7); ecchymosis (13) | | - | - |
| Sundus et al (2021) (41) | 412 | 412 | 10  erythematous eruptions (1), pseudo chilblain-like (2), maculopapular rash (5), livedo reticularis (2) | - | | - |
| Thuangtong et al (2021) (42) | 93 | 93 | 7  maculopapular rash (3), urticarial (2), petechiae (1), eczema (1) | - | | - |
| Yildiray et al (2021) (43) | 266 | 266 | 5  urticarial (3), vesicular (2) | - | | - |
| Dupont et al (2022) (44) | 2968 | 2968 | 30  urticarial eruption (4), maculopapular exanthema (6), papulovesicular exanthema (3), pernio-like (8), livedo (5), vasculitis (purpura-like) (4) | - | | - |
| Farhood et al (2022) (45) | 100 | 100 | 50  vesicular rash (4), maculopapular rash (6), diffuse urticaria (14), acrocyanosis (2), chilblain like (6), purpura (6), diffuse petechiae (12) | - | | - |
| Ghafoor et al (2022) (46) | 1026 | 1026 | 72  Maculopapular (27), urticarial (15), vesicular/pustular (10), papulosquamous (5), vascular (15) | - | | - |
| Mohammed et al (2022) (47) | 273 | 273 | 271  pseudo-chilblains (106), erythema (multiform–like eruptions, and not otherwise specified) (18), maculopapular rash (58), livedo/purpura/necrosis (11), urticarial (38), vesicular (40) | - | | - |
| Mostafa et al (2022) (48) | 38 | 38 | 23  urticaria (21), pseudochilblains (1), papulovesicular (1) | - | | - |
| Niyatiwatchanchai et al (2022) (49) | 105 | 105 | 13  rash (13) | - | | - |
| Pardal et al (2022) (50) | 45 | 45 | 20  maculopapular rash (13), urticarial rash (4), vesicular rash (2), livedo reticularis (2), purpura (1) |  | |  |
| Parmar et al (2022) (51) | 93 | 93 | 6  pseudo chilblain-like (2), purpuric/livedoid (5) | - | | - |
| Tatliparmak et al (2022) (52) | 192 | 192 | 8  urticaria (4), maculopapular eruption (4) | - | | - |
| Zengarini et al (2022) (53) | 1053 | 1053 | 0 | - | | - |
| Cestari et al (2023) (54) | 50 | 50 | 48  maculopapular (22), urticarial rash (6), pseudochilblains (2), papular-vesicular eruption (2), livedo/purpura/necrosis (16) | - | | - |

Abbreviations - NS: Not Specified.

**Supplementary Table 6.** Summary of Finding (SoF) Table showing the certainty of evidence of primary outcomes according to GRADE approach.

| **Certainty assessment** | | | | | | | **№ of patients** | | **Effect** | | **Certainty** |
| --- | --- | --- | --- | --- | --- | --- | --- | --- | --- | --- | --- |
| **№ of studies** | **Study design** | **Risk of bias** | **Inconsistency** | **Indirectness** | **Imprecision** | **Other considerations** | **Skin manifestation by sex** | **placebo** | **Relative (95% CI)** | **Absolute (95% CI)** |  |
| **Female vs Male in the in the occurrence of skin manifestations of COVID-19** | | | | | | | | | | | |
| 10 | observational studies | not serious | serious^a^ | not serious | serious^b^ | none | 205/1034 (19.8%) | 181/1415 (12.8%) | **OR 1.19** (0.77 to 1.83) | **21 more per 1000** (from 26 fewer to 84 more) | ⨁◯◯◯ Very low |
| **Mild/Moderate vs Severe/Critical COVID-19 in the in the occurrence of skin manifestations** | | | | | | | | | | | |
| 9 | observational studies | serious^c^ | very serious^d^ | not serious | very serious^e^ | strong association^f^ | 78/339 (23.0%) | 298/1469 (20.3%) | **OR 2.32** (0.77 to 7.00) | **168 more per 1000** (from 39 fewer to 438 more) | ⨁◯◯◯ Very low |

**CI:** Confidence Interval; **OR:** Odds Ratio

#### Explanations:

**a.** There is divergence in the estimation of the effect of the studies. But there is no significant p-value.

**b.** 95%CI = 0.77-1.83.

**c.** Several studies have not made it clear whether study participants were sampled appropriately, which may impact the selection of patients with different severity.

**d.** There is divergence in the estimation of the effect of the studies, I² = 82% and p<0.00001.

**e.** 95%CI = 0.77-7.00.

**f.** OR > 2.

## Supplementary Figures


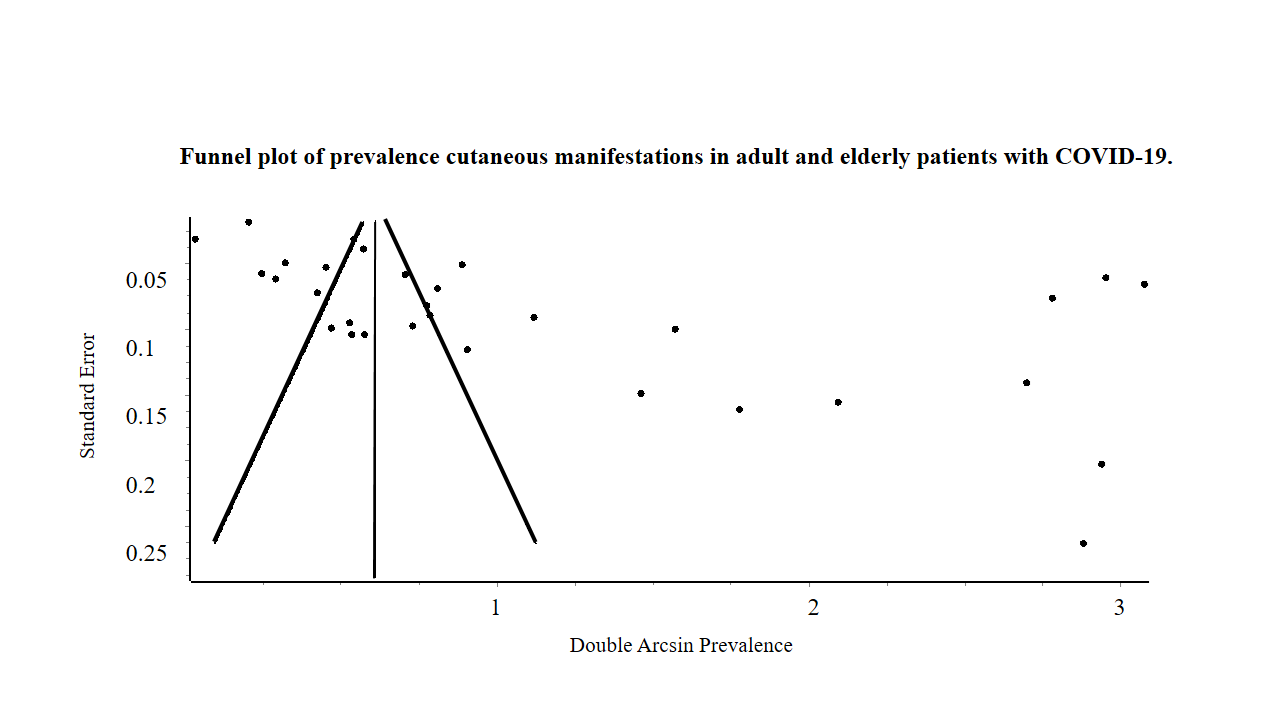


**Supplementary Figure 1.** Frequency of the skin manifestations in adult and elderly patients with COVID-19**:**  Funnel plot demonstrating the dispersion of studies included.


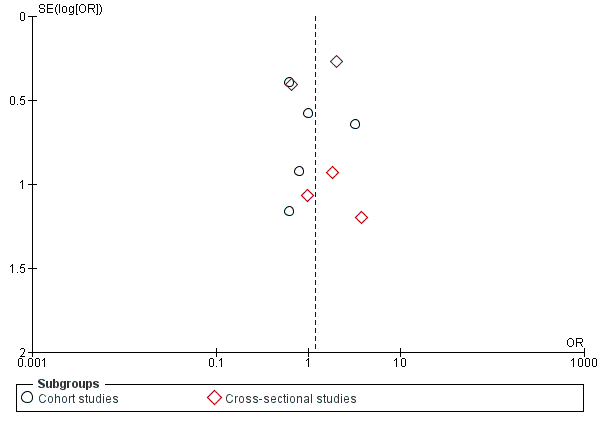


**Supplementary Figure 2.** Funnel plot of studies included in the meta-analysis of the association of skin manifestations of COVID-19 by sex.
